# Synthesis, structure, ionochromic and cytotoxic properties of new 2-(indolin-2-yl)-1,3-tropolones

**DOI:** 10.3762/bjoc.21.26

**Published:** 2025-02-17

**Authors:** Yurii A Sayapin, Eugeny A Gusakov, Inna O Tupaeva, Alexander D Dubonosov, Igor V Dorogan, Valery V Tkachev, Anna S Goncharova, Gennady V Shilov, Natalia S Kuznetsova, Svetlana Y Filippova, Tatyana A Krasnikova, Yanis A Boumber, Alexey Y Maksimov, Sergey M Aldoshin, Vladimir I Minkin

**Affiliations:** 1 Federal Research Centre the Southern Scientific Centre of the Russian Academy of Sciences, Rostov-on-Don 344006, Russian Federation; 2 Institute of Physical and Organic Chemistry, Southern Federal University, Rostov-on-Don 344090, Russian Federation; 3 Federal Research Center of Problems of Chemical Physics and Medical Chemistry of the Russian Academy of Sciences, Chernogolovka 142432, Russian Federation; 4 National Medical Research Center of Oncology, Rostov-on-Don 344037, Russian Federation; 5 Institute of Fundamental Medicine and Biology, Kazan (Volga Region) Federal University, Kazan 420008, Russian Federation; 6 O'Neil Comprehensive Cancer Center at University of Alabama at Birmingham, Department of Medicine, Section of Hematology/Oncology, Heersink School of Medicine, Birmingham, AL 35233, USA

**Keywords:** cytotoxic activity, fluorescence, *o*-chloranil, quantum chemical DFT calculations, 1,3-tropolones, X-ray diffraction

## Abstract

The acid-catalyzed reaction of benzo[*e*(*g*)] derivatives of 2,3,3-trimethylindolenines with *o*-chloranil leads to new 2-(benzo[*e*(*g*)]indolin-2-yl)-5,6,7-trichloro-1,3-tropolones and 2-(benzo[*e*(*g*)]indolin-2-yl)-4,5,6,7-tetrachloro-1,3-tropolones. Based on the results of PBE0/6-311+G(d,p) calculations, the structural and energetic characteristics of the tautomeric forms of the obtained 1,3-tropolones were determined. The structure of 2-(3,3-dimethyl-3*H*-benzo[*g*]indolin-2-yl)-5,6,7-trichloro-1,3-tropolone was determined by X-ray diffraction analysis. The compounds obtained are capable of switching emission at 420–440 nm and 476–530 nm upon successive exposure to CN^−^ and Hg^2+^ ions in an acetonitrile solution. 2-(1,1-Dimethyl-1*H*-benzo[*e*]indolin-2-yl)-5,6,7-trichloro-1,3-tropolone exhibited high in vitro cytotoxic activity against A431 skin cancer and H1299 lung cancer cell lines.

## Introduction

**Scheme 1:**
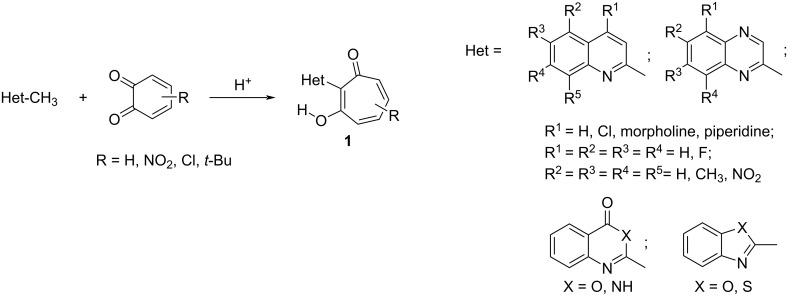
Synthesis of 2-hetaryl-substituted 1,3-tropolones **1**.

1,2-Benzoquinones represent unique building blocks for various classes of heterocyclic systems. Their structure depends on the reactivity of the initial heterocycles and 1,2-benzoquinones, as well as on the reaction conditions. In the series of 2-methylquinolines [1], 2-methylquinoxalines [2], 2-methylquinazolinones [3], 2-methylbenzoxazinones [4], and 2-methylbenzoxa(thia)zoles [5] the interaction with sterically hindered 1,2-benzoquinones and 3,4,5,6-tetrachloro-1,2-benzoquinone proceeds with the expansion of the *o*-quinone ring and results in 2-hetaryl-substituted 1,3-tropolones **1** (Scheme 1), which exhibit antibacterial [4] and cytotoxic activity [6–7].

Existing approaches to the synthesis of 1,3-tropolone derivatives have a number of drawbacks, namely, limited synthesis methodology and low yields of the target compounds [8–15]. Over the past decade, only a few reports have been published [16–17].

Indole derivatives, including those with conjugated aromatic and alicyclic rings, are of considerable interest because of their diverse biological activities (antibacterial, antimicrobial, anticancer, antidiabetic, etc.) [18–22]. For this reason, the synthetic goal of this work is to obtain heterocyclic indole compounds conjugated with a 1,3-tropolone moiety.

The variability of the products of acid-catalyzed reactions in the series of 2,3,3-trimethylindolenine with 1,2-benzoquinone derivatives depends on the nature of the substituents in the 1,2-benzoquinone. Thus, the interaction of 2,3,3-trimethylindolenine with 3,5-di(*tert*-butyl)-1,2-benzoquinone leads to the formation of indolo[1,2-*a*]indoline derivatives [23], while the presence of a nitro group in 4,6-di(*tert*-butyl)-3-nitro-1,2-benzoquinone the reaction with 2,3,3-trimethylindolenine leads to an *o*-quinone ring contraction and the formation of 2-azabicyclic products and pyridino[1,2-*a*]benzo[*e*]indol-10,11-diones [24]. *o*-Chloranil was found to be the most efficient 1,2-benzoquinone, which engages in *o*-quinone ring-expansion reactions with 2,3,3-trimethylindolenines to form hard-to-reach polychlorinated derivatives of 2-(indolin-2-yl)-1,3-tropolones [5]. Unlike the cross-aldol reaction of *o*-chloranil with methyl ketones [13–15], which is always accompanied by the removal of one of the chlorine atoms from the seven-membered ring, the acid-catalyzed reaction between methylene-active heterocyclic compounds and *o*-chloranil depends on the reaction conditions and can proceed with or without the inclusion of a dehydrochlorination stage and leads to 5,6,7-trichloro- or 4,5,6,7-tetrachlorotropones, respectively [5,25].

The present work reports the synthesis of new 2-(indolin-2-yl)-1,3-tropolones by the reaction of *o*-chloranil with benzoannelated derivatives of 2,3,3-trimethylindolenine, a comprehensive evaluation of the structure of the compounds obtained using quantum chemical methods, X-ray diffraction analysis, two-dimensional correlation NMR spectroscopy, as well as investigation of their ionochromic properties towards CN^−^ and Hg^2+^ ions and evaluation of in vitro cytotoxic activity against A431 skin cancer and H1299 lung cancer cell lines.

## Results and Discussion

**Scheme 2:**
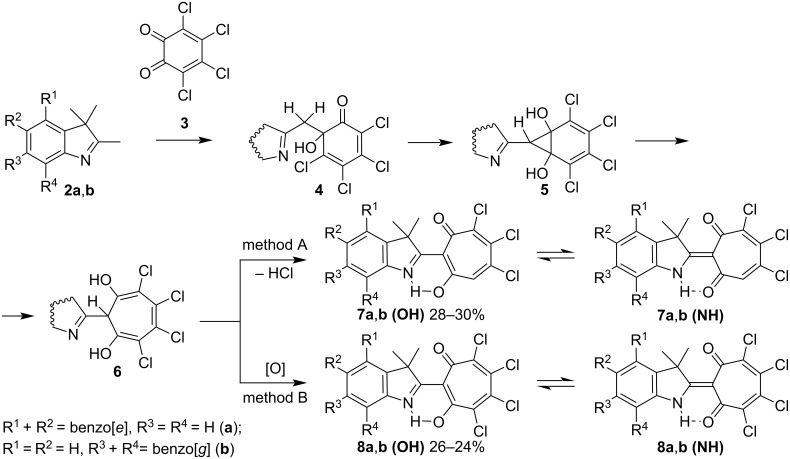
Synthesis of 1,3-tropolones **7a**,**b** and **8a**,**b**. Reagents and conditions: method A: dioxane, reflux; method B: AcOH, 40–50 °C.

We found that boiling of equimolar amounts of benzannelated 2,3,3-trimethylindolenines **2a**,**b** and *o*-chloranil (**3**) (method A) in dioxane leads to the formation of trichlorosubstituted 1,3-tropolones **7a**,**b** as the main reaction products (Scheme 2). Heating **2a**,**b** in acetic acid with a two-fold excess of *o*-chloranil (**3**) (method B) is accompanied by the formation of tetrachlorosubstituted 1,3-tropolones **8a**,**b** (Scheme 2 and Supporting Information File 1).

As shown in Scheme 2, in the initial step, the aldol condensation of 2,3,3-trimethylindolenine **2** with *o*-chloranil (**3**) leads to the intermediate compounds, 6-(2-hetarylmethylene)-6-hydroxy-2,4-cyclohexadien-1-ones **4**. Such intermediates were isolated preparatively and structurally characterized in the reactions of 2-methylquinolines with 3,5-di-(*tert*-butyl)-1,2-benzoquinone [1] and benzophenones with *o*-chloranil [15]. The norcaradiene derivatives **5**, formed in the next step by the intramolecular cyclization reaction of **4**, undergo thermal isomerization into 2,3-dihydrotropolones **6**. The formation of 2-(indolin-2-yl)-5,6,7-trichloro-1,3-tropolones **7a**,**b** is accompanied by dehydrochlorination of **6** upon boiling in dioxane according to method A. One of the conditions for carrying out the reaction according to method B is the use of a two-fold excess of quinone **3**. Oxidation of dihydrotropolones **6** with an excess of quinone **3** leads to the formation of 2-(indolin-2-yl)-4,5,6,7-tetrachloro-1,3-tropolones **8a**,**b** as final products. The detailed reaction mechanism in acetic acid solution was studied by the PBE0/6-311+G(d,p) method on the example of the interaction of 2-methylquinolines and 2-methylbenzazoles with 1,2-benzoquinone and *o*-chloroanil, respectively [1,25].

The structures of compounds **7** and **8** obtained by methods A and B were confirmed by ^1^H NMR, IR spectroscopy and mass spectrometry (Supporting Information File 2). A distinctive feature of the ^1^H NMR spectra of **7** in CDCl_3_ is the signal of the tropolone ring proton, which appears at 6.9 ppm. A characteristic specificity of the ^1^H NMR spectra of compounds **7** and **8** is the presence of signals of hydroxy group protons forming a strong hydrogen bond with the indoline nitrogen atom, which closes the six-membered chelate cycle. These signals are observed in the weak field at 15.2–15.8 ppm for **7a**,**b** and 14.3–14.8 ppm for **8a**,**b**, respectively, as a broadened singlet peak.

**Figure 1:**
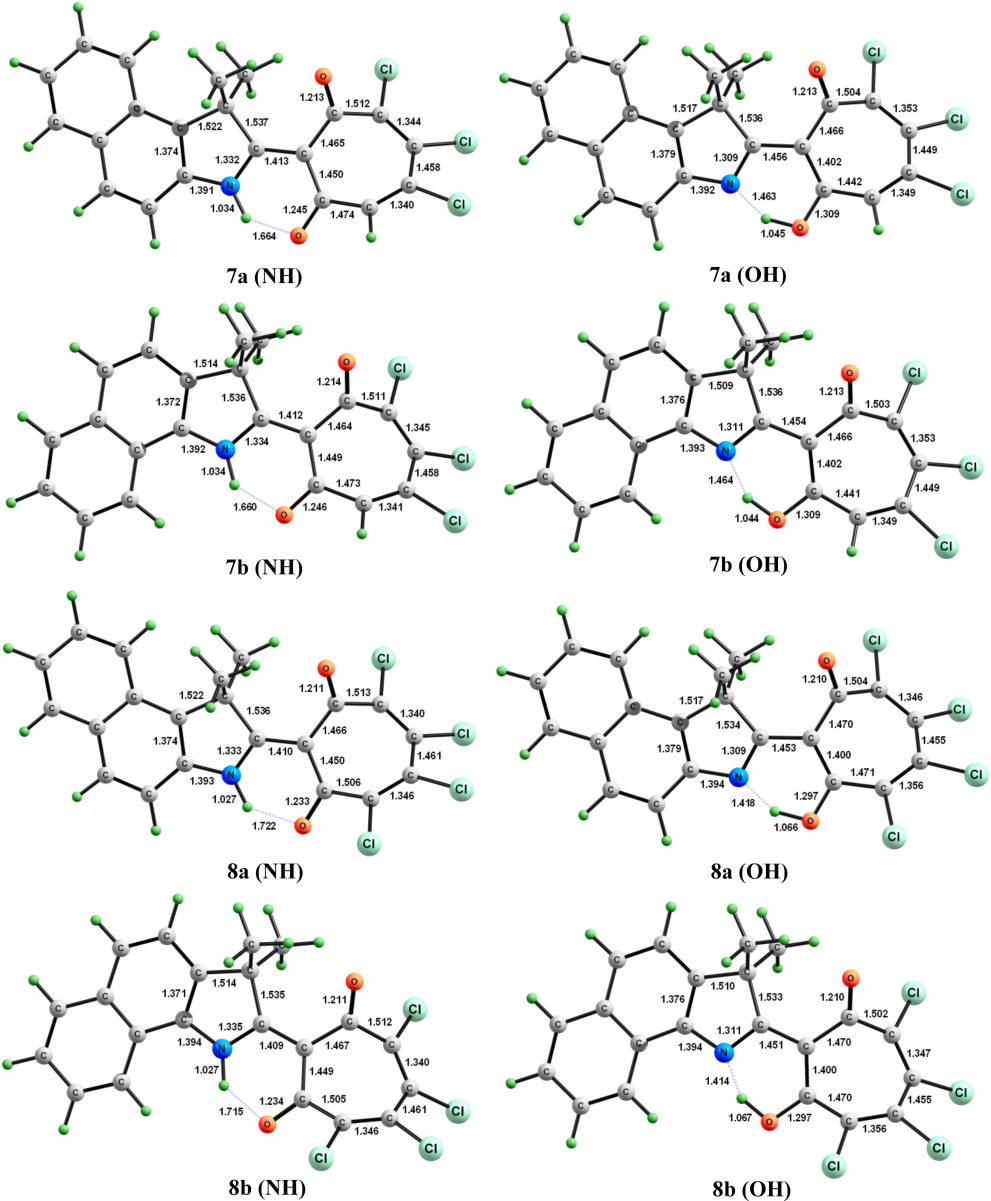
Structural characteristics of (**NH**) and (**OH**) tautomeric forms of compounds **7** and **8** in the gas phase according to PBE0/6-311+G(d,p) calculations. Bond lengths are given in Ångstrom.

A dynamic equilibrium of tautomeric forms **7**, **8** (**OH**)–**7**, **8** (**NH**) exists in solution, which can be detected by the broadening of the hydroxy group proton signal in the ^1^H NMR spectrum (Scheme 2). The structures of tautomeric forms of **7a**, **7b**, **8a**, **8b** and their energy characteristics were calculated by PBE0/6-311+G(d,p) method in the gas phase and polar solvent (DMSO) (Table 1, Figure 1 and Supporting Information File 3). According to the obtained data, the (**NH**) isomers of **7** and **8** are thermodynamically more stable than the corresponding (**OH**) forms. Increasing the polarity of the medium additionally enhances their stabilization. The introduction of an additional acceptor substituent to the tropolone moiety has a similar effect. In addition, compared to compounds **7a**,**b**, compounds **8a**,**b** show a marked increase in the intramolecular hydrogen bond length NH···O of the (**NH**) isomers and a decrease in the magnitude of hydrogen bond N···HO of the (**OH**) forms (Figure 1). On the other hand, the position of the benzoannelated fragment does not significantly affect the structural and energetic parameters of the studied compounds.

**Table 1:** Total energies with zero-point energy correction (*E*_tot_ + ZPE, a.u.) and relative energies (Δ*E*, kcal/mol) of (**NH**) and (**OH**) isomers of compounds **7** and **8** calculated by the PBE0/6-311+G(d,p) method in the gas phase (gas) and DMSO solution (sol).

compound	*E*_tot_ + ZPE(gas)	Δ*E*_gas_	*E*_tot_ + ZPE(sol)	Δ*E*_sol_

**7a** (**NH**)	−2392.806710	0	−2392.818223	0
**7a** (**OH**)	−2392.803386	2.1	−2392.812918	3.3
**7b** (**NH**)	−2392.809986	0	−2392.821048	0
**7b** (**OH**)	−2392.806819	2.0	−2392.816317	3.0
**8a** (**NH**)	−2852.269500	0	−2852.281433	0
**8a** (**OH**)	−2852.263504	3.8	–	–
**8b** (**NH**)	−2852.272654	0	−2852.284199	0
**8b** (**OH**)	−2852.266797	3.7	–	–

The difference in the energy characteristics of the (**NH**) and (**OH**) tautomeric forms of compounds **7a**, **7b**, **8a**, and **8b** in the polar solvent and the gas phase is small (2.0–3.8 kcal/mol), and probably the nature of the solvent can influence the shift of the equilibrium towards one of the tautomeric forms.

To reveal the predominance of either the (**NH**) or (**OH**) tautomeric form of compounds **7a**, **7b**, **8a**, and **8b** in solution depending on the nature of the solvent, we carried out a detailed study using compound **7a**. A complete signal assignment of ^1^H and ^13^C NMR spectra was carried out, based on characteristic values of chemical shifts and cross-peak analysis in two-dimensional spectra of ^1^H,^1^H COSY correlations, as well as ^1^H,^13^C correlations HSQC, HMBC, and ^1^H,^15^N HMBC spectra in DMSO-*d*_6_ and CDCl_3_ (Supporting Information File 2).

**Figure 2:**
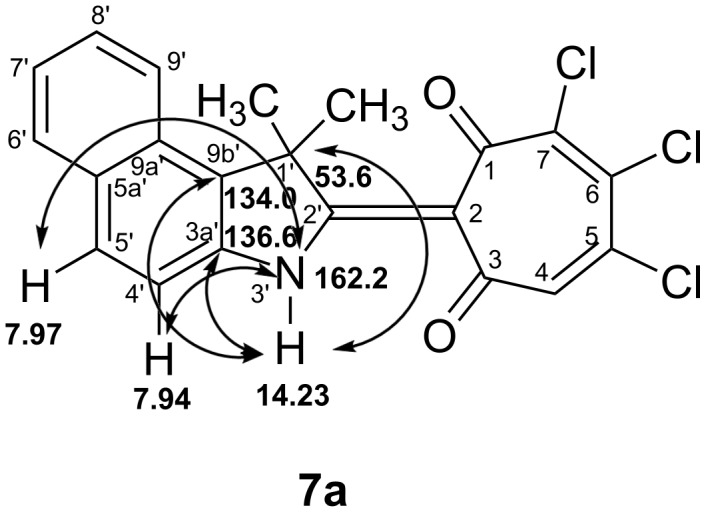
Scheme of HMBC correlations of compound **7a** in DMSO-*d*_6_.

In the ^1^H NMR spectrum of **7a** in DMSO-*d*_6_, the signal of the weak field proton is shifted to a stronger field of 14.23 ppm and appears as a narrow singlet (Supporting Information File 2, Figure S17). Analysis of two-dimensional correlation spectra of heteronuclear NMR spectroscopy shows that compound **7a** in DMSO-*d*_6_ solution exists in the (**NH**) tautomeric form. Thus, in the ^1^H,^15^N HMBC spectrum of **7a** there are cross peaks of the indolenine nitrogen atom at 162.2 ppm with a weak field proton at δ_H_ 14.23 ppm, as well as aromatic protons H(4') and H(5') with δ_H_ 7.94 ppm and 7.97 ppm, respectively (Supporting Information File 2, Figure S19). In the two-dimensional ^1^H,^13^C HMBC spectrum of **7a**, a correlation of the NH proton with the bridging carbon atoms C(3a'), C(9b'), and the quaternary carbon atom C(1') of the indolenine fragment is observed with δ_H_ 136.6 ppm, 134.0 ppm, and 53.6 ppm, respectively (Supporting Information File 2, Figure S20). The most important HMBC correlations for the analysis of structure **7a** are schematically depicted in Figure 2.

At the same time, in the ^1^H,^15^N HMBC spectra of **7a** in CDCl_3_, there are no cross-peaks of the proton in the region of δ_H_ 15.15 ppm with the nitrogen atom of the indolenine fragment at 162.7 ppm, and in the ^1^H,^13^C HMBC spectrum, no correlations of the down-field proton with the carbon atoms of the indolenine fragment are observed (Supporting Information File 2, Figures S21 and S22). Thus, in CDCl_3_, compound **7a** is predominantly in the (**OH**) tautomeric form.

**Figure 3:**
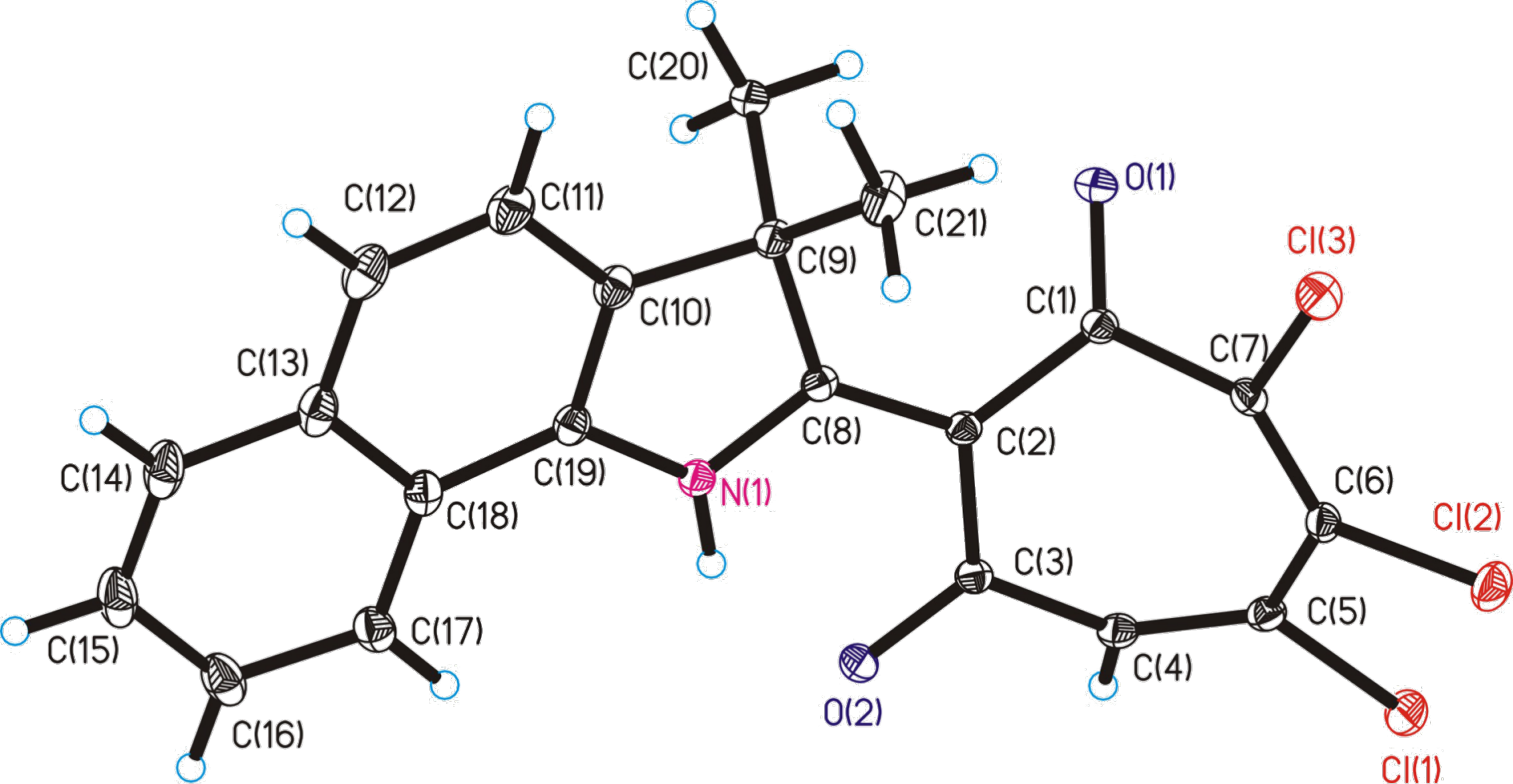
Molecular structure of 2-(3,3-dimethyl-3*H*-benzo[*g*]indolin-2-yl)-5,6,7-trichloro-1,3-tropolone (**7b**).

The structure of 2-(3,3-dimethyl-3*H*-benzo[*g*]indolin-2-yl)-5,6,7-trichloro-1,3-tropolone (**7b**) was established by X-ray diffraction analysis (Figure 3). The main distances and angles are summarized in Supporting Information File 3.

**Figure 4:**
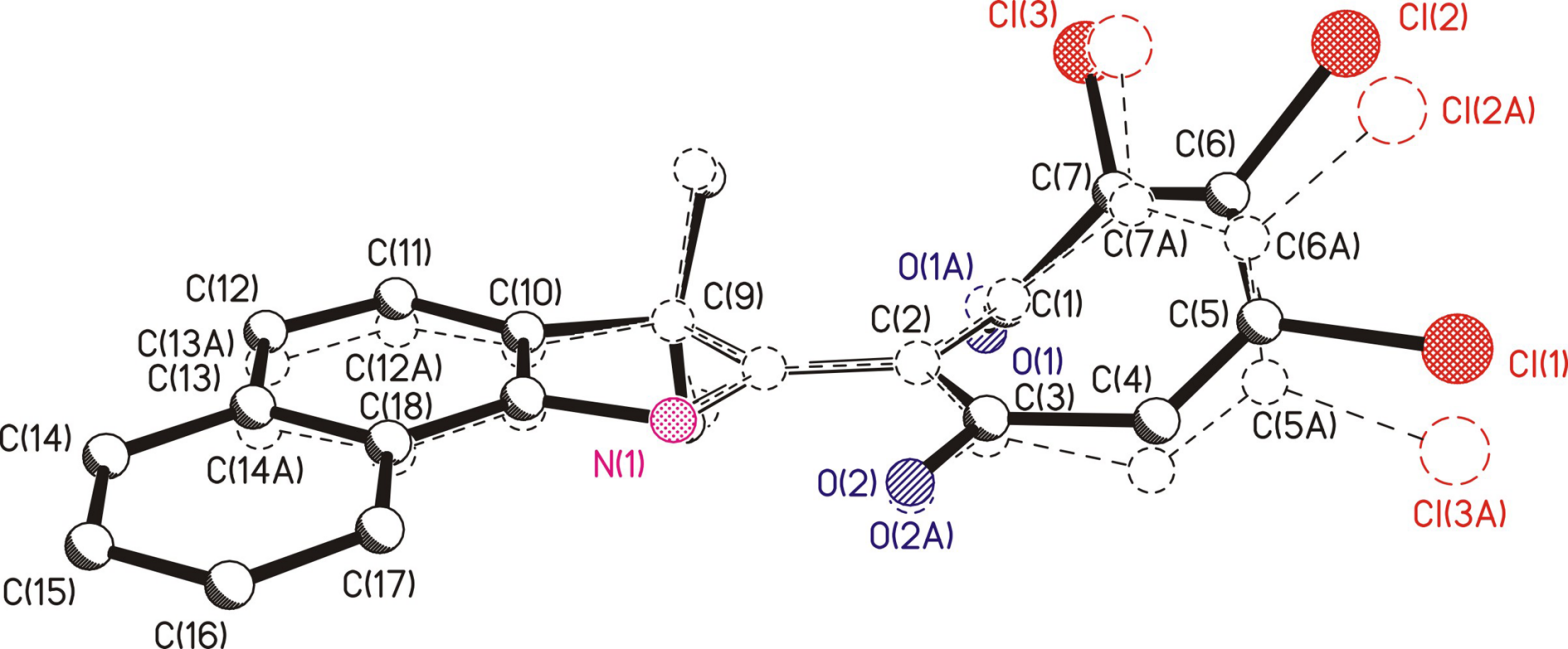
Result of matching structures of **7b** (solid lines) and 2-(3,3-dimethylindolin-2-yl)-5,6,7-trichloro-1,3-tropolone (dashed lines) at the positions of N(1), C(2), and C(9) atoms.

To compare the structural characteristics, we combined the molecule **7b** (solid lines) and the previously obtained 2-(3,3-dimethylindolin-2-yl)-5,6,7-trichloro-1,3-tropolone [5] (dashed lines) at the positions of N(1), C(2) and C(9) atoms (hydrogen atoms removed) (Figure 4).

While maintaining the general configuration of the molecular framework, there are minor differences in the details of their structures, manifested in the observed distances between the atoms C12)···C(13A) = 0.31 Å, Cl(2)···Cl(2A) = 0.76 Å and in the geometric parameters of the tropolone rings. The angle between the planes C(2)C(3)C(6)C(7) and C(1)C(2)C(7) is equal to 42.9° (42.1°) (here and below, when comparing geometric values, similar values are given in parentheses for the previously obtained 2-(indolin-2-yl)-1,3-tropolone)). The angle between the planes C(2)C(3)C(6)C(7) and (3)C(4)C(5)C(6) is equal to 27.6° (34°). In compound **7b**, as in 2-(indolin-2-yl)-1,3-tropolone, an intramolecular hydrogen bond N(1)–H(1)···O(2) was realized with parameters: distances N–H = 0.86 (0.84) Å, H···O = 1.79 (1.83) Å, and N···O = 2.513 (2.518) Å, angle N–H–O 140.4° (138.0°).

**Figure 5:**
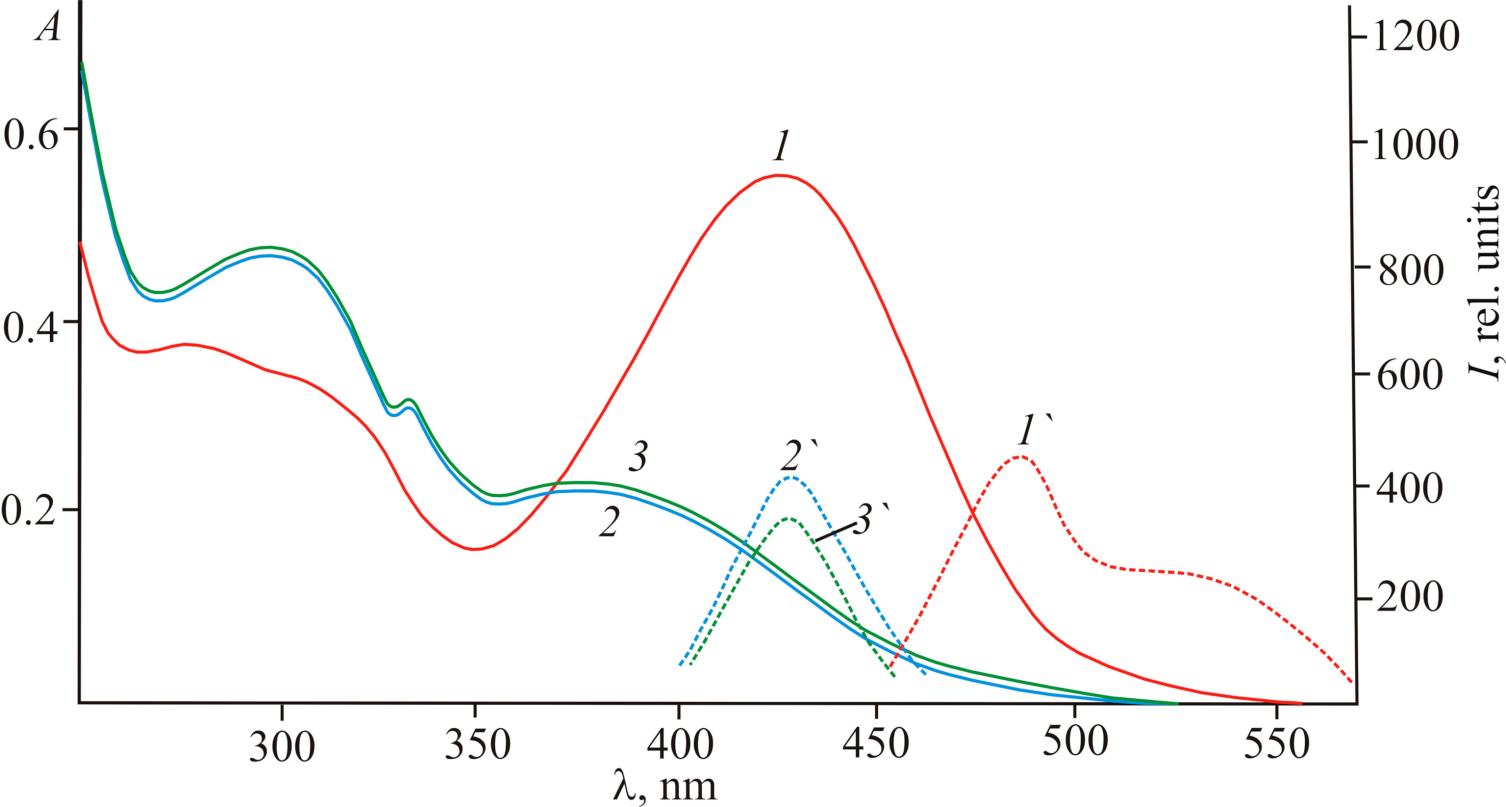
Absorption and emission spectra of compound **8b** in acetonitrile before (*1,1’*) (*c* 2.5 × 10^−5^ mol L^–1^) and after the addition of CN^−^ (*2,2’*) and F^−^ (*3,3’*) ions (*c* 5.0 × 10^−5^ mol L^−1^).

The electronic absorption spectra of compounds **7a**,**b** and **8a**,**b** in acetonitrile have long wavelength bands in the region of 425–432 nm (Table 2). They exhibit dual-maxima fluorescence at 476–530 nm with normal and large Stokes shift values (Table 2, Figure 5).

**Table 2:** Absorption and fluorescence spectral data of compounds **7a**,**b** and **8a**,**b** in CH_3_CN.^a^

compound	absorption, λ_max_, nm (ε, L mol^−1^ cm^−1^)	fluorescence, λ_max_, nm (*I*_fl_, rel. units)	Stokes shift, (Δν_fl_, cm^−1^)

**7a**	276 (17200), 300 (18400), 427 (20400)	485 (260), 520 (180)	2800, 4200
**7b**	292 (14000), 432 (19100)	495 (220), 523 (190)	2950, 4050
**8a**	300 (14800), 310 (15200), 418 (20000)	476 (420), 520 (190)	2900, 4700
**8b**	300 (14400), 425 (21900)	491(480), 530 (250)	3150, 4650

^a^*I*_fl_ – the relative fluorescence intensity; *c* 2.5 × 10^−5^ mol L^−1^, λ_ex_ 417 nm, PMT voltage 940 V.

This is consistent with the above conclusion about the existence of tautomeric equilibrium **7**,**8** (**OH**)–**7**,**8** (**NH**) in solutions based on NMR data and DFT quantum chemical calculations (Scheme 2). The emission with a larger Stokes shift appears to correspond to the **7**,**8** (**OH**) form and is caused by the excited-state intramolecular proton transfer (ESIPT) process due to intramolecular O→N proton migration in the singlet excited state [26–27].

**Scheme 3:**
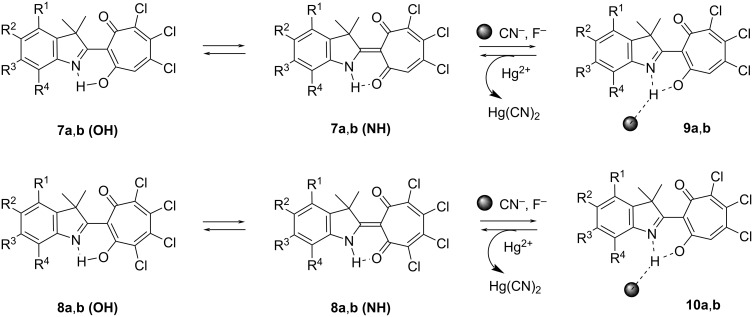
Possible binding mode of **7** and **8** with CN^−^ and F^−^.

The ionochromic sensitivity of compounds **7a**,**b** and **8a**,**b** to anions was investigated in acetonitrile upon addition of tetra-*n*-butylammonium salts (TBAX: F, Cl, Br, I, CN). Exclusively cyanide and fluoride anions lead to a naked-eye effect due to a change of the solution’s colour from yellow-orange to pale yellow (Figure 5). At the same time, a new fluorescence band appears at 420–440 nm. The Stokes shifts of fluorescence decrease to normal values of 1800–2000 cm^−1^, indicating a complete inhibition of the ESIPT process. These spectral transformations are usually caused by the formation of a strong N–H(O–H)···CN^−^ (F^−^) hydrogen bond up to deprotonation [28]. Using the principle of “relay recognition” [29], we investigated the sensitivity of in situ obtained complexes **9** and **10** with CN^−^ to different cations. It appeared that the addition of an equivalent amount of Hg(ClO_4_)_2_ to an acetonitrile solution selectively and completely restores the initial absorption and fluorescence spectra (Scheme 3).

Thus, the obtained compounds represent molecular switches of optical and fluorescent properties under sequential addition of CN^−^ and Hg^2+^ ions and the transformation cycle presented in Scheme 3 can be repeated at least 5–6 times.

**Figure 6:**
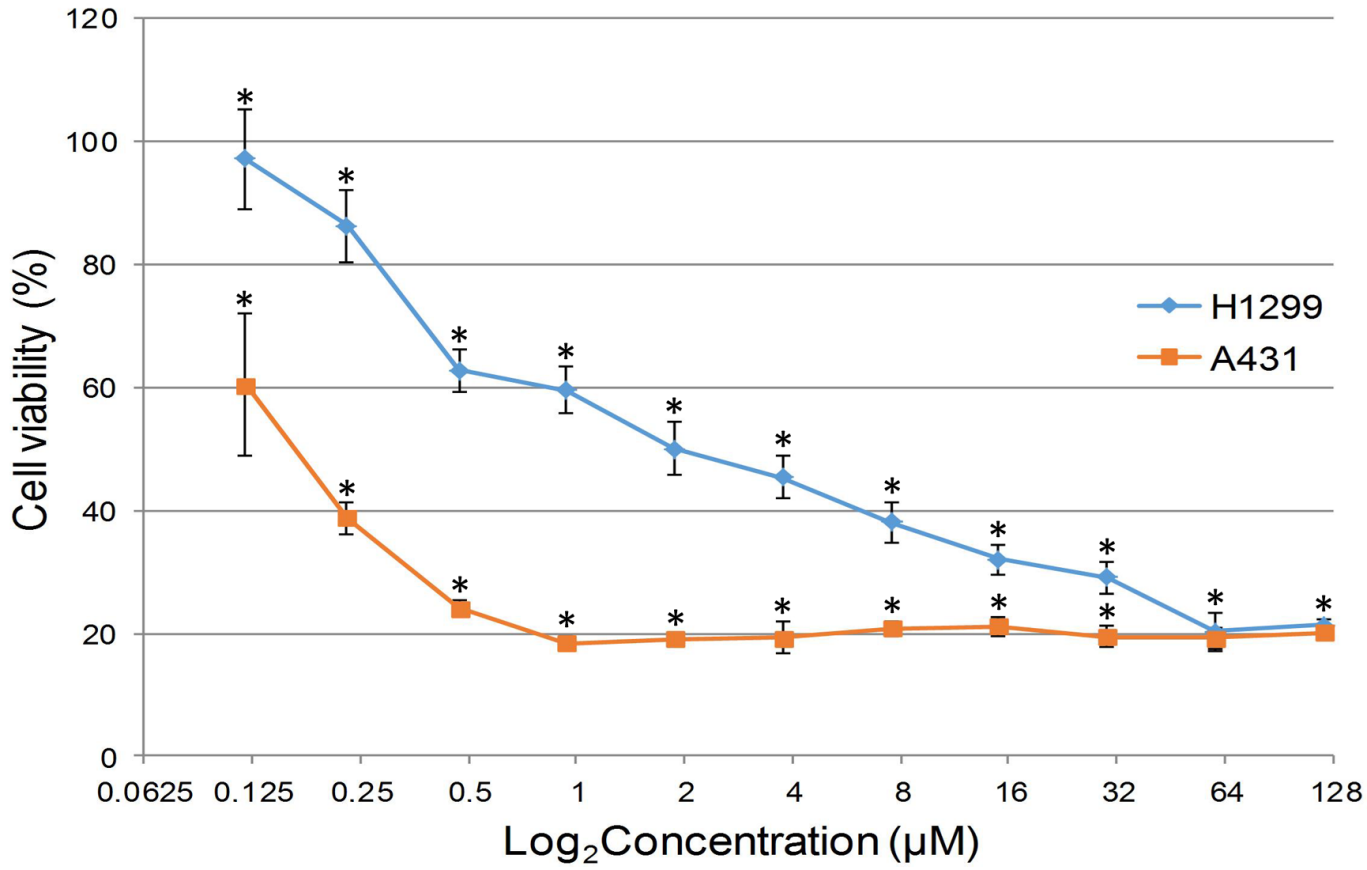
Dose–response curves for H1299 and A431 cells treated with compound **7a** for 24 h. *Significant difference compared to control samples, *p* < 0.05.

The resulting compounds **7b** and **8a**,**b** have extremely low water solubility, which makes it impossible to prepare concentrations sufficient for studying cellular cytotoxicity. In this regard, 1,3-tropolone **7a**, which has an acceptable level of solubility in water, was used for biological testing. The in vitro cytotoxic activity was investigated with A431 skin cancer and H1299 lung cancer cell lines. A standard MTT assay was used to determine the anticancer activity of compound **7a** against these cultures (Figure 6). The viability test is an important method to determine which new compounds are able to target cancer cells. The results of this test (IC_50_ value ± 95% confidence interval) allow us to evaluate the possibility of carrying out subsequent stages of research and selecting promising compounds for the further development of anticancer drugs [30].

The study showed that the IC_50_ inhibitory concentration of 2-(1,1-dimethyl-1*H*-benzo[*e*]indolin-2-yl)-5,6,7-trichloro-1,3-tropolone (**7a**) against the A431 skin cancer cell culture, at which the number of live cells was reduced by 50%, was 0.172 ± 0.029 μM. For the H1299 culture, this value was 2.18 ± 0.7 μM. In comparison, the IC_50_ value of the standard anticancer drug cisplatin at 24 hour incubation is 137 ± 12 μM for the A431 culture [31] and 34.9 μM for the H1299 culture [32], which is significantly higher than the values obtained for compound **7a**.

## Conclusion

In summary, new 2-(benzo[*e*(*g*)]indolin-2-yl)-5,6,7-trichloro-1,3-tropolones **7a**,**b** and 2-(benzo[*e*(*g*)]indolin-2-yl)-4,5,6,7-tetrachloro-1,3-tropolones **8a**,**b** were obtained by acid-catalyzed reactions of benzo[*e*(*g*)] derivatives of 2,3,3-trimethylindolenines with *o*-chloranil. The energetic characteristics of the tautomeric forms of 1,3-tropolones **7** and **8** were determined by PBE0/6-3-311+G(d,p) calculations, and the structure of 2-(3,3-dimethyl-3*H*-benzo[*g*]indolin-2-yl)-5,6,7-trichloro-1,3-tropolone (**7b**) was established by X-ray diffraction analysis. Using two-dimensional correlation NMR spectroscopy methods, it was shown that the nature of the solvent significantly affects the equilibrium of the tautomeric forms of the 1,3-tropolones. The obtained compounds represent molecular switches of optical and fluorescent properties under sequential addition of CN^−^ and Hg^2+^ ions. 2-(1,1-Dimethyl-1*H*-benzo[*e*]indolin-2-yl)-5,6,7-trichloro-1,3-tropolone (**7a**) was found to exhibit high in vitro cytotoxic activity against A431 skin cancer and H1299 lung cancer cell lines. In addition, the IC_50_ values of compound **7a** are significantly lower than the IC_50_ values of cisplastin, which is widely used in the therapy of tumors, including lung cancer.

## Experimental

### General

^1^Н, ^13^С and ^15^N NMR spectra were obtained on the integrated analytical LC–SPE–NMR–MS system AVANCE-600 (Bruker) (600 MHz, ^1^Н; 150 MHz, ^13^С; 60 MHz, ^15^N) at 20 °C in CDCl_3_ and DMSO-*d*_6_*.* The signals were referenced to the signals of residual proton signals of corresponding deutero-solvents. IR spectra were recorded on a Varian Excalibur 3100 FTIR instrument using the attenuated total internal reflection technique. Mass spectra were obtained on a Finnigan Mat Incos 50 spectrometer. Electronic absorption spectra were obtained on a Varian Cary 100 spectrophotometer. Emission spectra were recorded on a Varian Cary Eclipse spectrofluorimeter. Acetonitrile of spectroscopic grade (Aldrich), previously purified by distillation, and tetra-*n*-butylammonium salts (TBAX: F, Cl, Br, I, CN) (Aldrich) were used to prepare the solutions. Spectral fluorescent experiments were performed using quartz cells (*l*_cuvette_ 1.0 cm, volume *V* 2 mL). Stock solutions of compounds **7** or **8** (*c* 5.0 × 10^−5^ mol L^−1^) and tetrabutylammonium salts (*c* 1.0 × 10^−4^ mol L^−1^) in acetonitrile were used. The corresponding solution (1 mL) and the tetra-*n*-butylammonium salt (1 mL) solution were mixed directly in the cell and thoroughly stirred. Hence, the working concentrations of compounds **7** or **8** and anions was 2.5 × 10^−5^ mol L^−1^ and 5.0 × 10^−5^ mol L^−1^, respectively. All spectral experiments were performed at room temperature (23 °C). Chromatography was carried out on columns filled with Al_2_O_3_ of II–III degree of activity according to Brockmann. Melting points were determined on a Fisher-Johns melting point apparatus. 1,1,2-Trimethyl-1*H*-benzo[*e*]indolenine (**2a**, 98%, Alfa Aesar), 2,3,3-trimethyl-3*H*-benzo[*g*]indolenine (**2b**, 98%, Alfa Aesar) and tetrachloro-*o*-benzoquinone (*o*-chloranil, **3**) (97%, ACROS organics) were used as the starting reagents. NMR and IR spectra were recorded on the equipment of the Center for Collective Usage “Molecular Spectroscopy” of Southern Federal University.

### X-ray diffraction study

The unit cell parameters and reflection intensities for compound **7b** (a three-dimensional set) were measured on an Xcalibur EOS autodiffractometer (Mo Kα irradiation, graphite monochromator, 150 K). Orange monoclinic crystals, chemical formula C_21_H_14_NO_2_Cl_3_, *М* = 418.68, *a* = 11.4480(3), *b* = 7.4883(2), *с* = 21.7720(6) Å, β = 102.590(3)°, *V* = 1821.55(9) Å^3^, *Z* = 4, ρ_calc_ = 1.527 g cm^−3^, μ(Мо Кα) = 0.520 mm^−1^, *P*2_1_/*c* space group. Intensities of 15441 reflections were measured in the reciprocal space (2θ ≤ 64.14°) using the ω/2θ-scanning method from a single crystal with dimensions of 0.31 × 0.25 × 0.20 mm. An empirical accounting of absorption was carried out using the Multiscan procedure. After exclusion of systematically cancelled reflections and averaging intensities of equivalent reflections, the working array of measured *F*^2^*(hkl)* and σ*(F*^2^*)* reflections contained 6352 independent reflections, 5089 of which with *F*^2^ > 4σ*(F*^2^*).* The structure was solved with the direct method and was refined by the full-matrix least-squares procedure (LSP) with respect to *F*^2^ in anisotropic approximation for non-hydrogen atoms (hydrogen atoms isotropic) using the SHELXTL program. In the crystal structure, most of the H atoms are localized in the Fourier synthesis of the difference electron density, then the coordinates and isotropic thermal parameters of all H atoms were calculated by the LSP using the “rider” model [33]. The absolute shifts of all 247 varied structure parameters were less than 0.001 σ, the final value of the factor *R*_1_ = 0.0385. The CIF file containing atomic coordinates, full tables of bond lengths, bond angles, and thermal parameters of compound **7b** have been deposited at the Cambridge Crystallographic Data Centre (CCDC 2040907) and can be obtained upon request on the website: https://www.ccdc.cam.ac.uk/data_request/cif.

### Computational methods

Quantum chemical calculations were performed using the Gaussian 09 software package [34] with hybrid functional PBE0 [35–36] and 6-311+G (d,p) basis set. Solvent effects were modeled by using polarizable continuum model (PCM) [37] within the integral equation formalism (IEFPCM) [38].

### Biological experiments

The MTT colorimetric test for cell viability assessment is based on the reduction by NADPH-H-dependent cellular oxidoreductase enzymes of the tetrazolium dye 3-(4,5-dimethylthiazol-2-yl)-2,5-diphenyltetrazolium bromide, which has yellow color, into violet-blue formazan, with absorption maximum in the range of 540–560 nm. The optical density of the solution in this wavelength range is an indirect indicator of the number of live cells in the culture. The cytotoxic activity of the test substance was determined by the decrease in optical density of experimental samples compared to control samples [39].

A431 and H1299 cells were seeded into 96-well plates at 15000/well in DMEM medium supplemented with 10% EFV and cultured under standard conditions at 5.0% CO_2_ and 37 °C. The next day, the medium was replaced and test compound **7a** was added in a series of two-fold dilutions from 0.12 μM to 120 μM. In the control wells, the medium was replaced without adding the compound **7a**. The cells were then incubated under the same standard conditions for 24 h, after which the medium was replaced and 10% MTT solution in DMEM medium supplemented with 10% EFV was added and incubated for another 2 h under CO_2_ incubator conditions. At the end of cultivation, the medium with MTT was completely removed from the wells, and the formed formazan crystals were dissolved in DMSO and the optical density of the resulting solution was measured at 540 nm. The level of cytotoxic activity was determined by the change in optical density at 540 nm in wells treated with the compound **7a** or cisplatin compared to control wells. The experiment was carried out in three biological replicates with 6 technical replicates in each. Results were analyzed using one-way ANOVA followed by Tukey’s post hoc test of significance. IC_50_ values were determined in the RStudio development environment using the DRC software package [40].

## Supporting Information

File 1 Experimental procedures and characterization data for all novel compounds **7a**, **7b**, **8a** and **8b**.

File 2 ^1^H, ^13^C NMR, IR and HRMS spectra of all novel compounds.

File 3 X-ray analysis data of **7b** and DFT quantum chemical calculations for **7a**, **7b**, **8a** and **8b**.

File 4 Crystallographic information file for compound **7b**.

## Data Availability

All data that supports the findings of this study is available in the published article and/or the supporting information of this article.
