# Supplementary material for: Synthesis, structure, ionochromic and cytotoxic properties of new 2-(indolin-2-yl)-1,3-tropolones
Source: Beilstein J Org Chem. 2025 Feb 17;21:358–68. doi: 10.3762/bjoc.21.26 (PMC11849549; doi:10.3762/bjoc.21.26)
Supplement: File 1 — Experimental procedures and characterization data for all novel compounds 7a, 7b, 8a and 8b. [file Beilstein_J_Org_Chem-21-358-s001.pdf]

BEILSTEIN JOURNAL OF ORGANIC CHEMISTRY

Supporting Information

for

Synthesis, structure, ionochromic and cytotoxic properties of new 2-(indolin-2-yl)-1,3-tropolones

Yurii A. Sayapin, Eugeny A. Gusakov, Inna O. Tupaeva, Alexander D. Dubonosov, Igor V. Dorogan, Valery V. Tkachev, Anna S. Goncharova, Gennady V. Shilov, Natalia S. Kuznetsova, Svetlana Y. Filippova, Tatyana A. Krasnikova, Yanis A. Boumber, Alexey Y. Maksimov, Sergey M. Aldoshin and Vladimir I. Minkin

Beilstein J. Org. Chem. **2025,** *21,* 358–368. doi:10.3762/bjoc.21.26

Experimental procedures and characterization data for all novel compounds 7a, 7b, 8a and 8b

License and Terms: This is a supporting information file under the terms of the Creative Commons Attribution License (https://creativecommons.org/licenses/by/4.0). Please note that the reuse, redistribution and reproduction in particular requires that the author(s) and source are credited and that individual graphics may be subject to special legal provisions.
The license is subject to the *Beilstein Journal of Organic Chemistry* terms and conditions: (https://www.beilstein-journals.org/bjoc/terms)

Synthesis

2-(1,1-Dimethyl-1*H*-benzo[*e*]indolin-2-yl)-5,6,7- trichloro-1,3-tropolone (**7a**).

A solution of 1,1,2-trimethyl-1*H*-benzo[*e*]indoline (**2a**, 1.05 g, 5 mmol) and *o*-chloranil (**3**, 1.23 g, 5 mmol) in dioxane (10 mL) was refluxed for 1.5 h. The solvent was removed *in vacuo* and the residue was dissolved in methylene chloride. The solution was purified by silica gel column chromatography with hexane/CH_2Cl_2 1:2. The yellow-orange fraction of **7a** with R_f 0.4 was collected and recrystallized from *n*-hexane. Orange crystals. Yield 30%, mp 164-165 °C (*n*-hexane). ^{1}H NMR (DMSO-d_6) δ (ppm), *J*/Hz: 1.85 (s, 6H, 2Me), 7.10 (s, 1H, H^4), 7.51 (t, 1H, H_{Ar}, J = 7.8), 7.63 (t, 1H, H_{Ar}, J = 7.8), 7.94 (d, 1H, H_{Ar}, J = 8.4), 7.97 (d, 1H, H_{Ar}, J = 8.4) 8.02 (d, 1H, H_{Ar}, J = 7.8), 8.17 (d, 1H, H_{Ar}, J = 8.4), 14.23 (s, 1H, OH). ^{13}C NMR (DMSO-d_6) δ (ppm): 22.9, 53.6, 111.7, 114.2, 122.4, 125.0, 127.0, 127.4, 128.0, 129.8, 129.8, 130.6, 132.0, 134.0, 134.4, 134.8, 136.6, 178.3, 182.5, 182.7. ^{1}H NMR ($CDCl_3$) δ (ppm), *J*/Hz: 1.98 (s, 6H, 2Me), 6.92 (s, 1H, H^4), 7.45 (d, 1H, H_{Ar}, J = 9), 7.51 (t, 1H, H_{Ar}, J = 7.8), 7.64 (t, 1H, H_{Ar}, J = 8.4), 7.89 (d, 1H, H_{Ar}, J = 8.4), 7.96 (d, 1H, H_{Ar}, J = 8.4), 8.09 (d, 1H, H_{Ar}, J = 8.4), 15.15 (br. s, 1H, OH). ^{13}C NMR ($CDCl_3$) δ (ppm): 14.1, 23.4, 31.6, 54.6, 112.7, 122.5, 125.3, 127.6, 127.9, 130.1, 130.3, 132.6, 133.5, 135.0, 135.9, 180.7. IR (ν, cm^{-1}): 3040, 2975, 2934, 2867, 1736, 1638, 1536, 1520, 1494, 1466, 1442, 1399, 1372, 1364, 1323, 1261, 1215, 1207, 1174, 1082, 1057, 945, 907, 874, 856, 815, 788, 763, 748, 689. ESI-MS, *m/z* (rel. int.): 419 (44) [M^+], 417 (44), 389 (66), 378 (27), 374 (82), 353 (17), 339 (60), 304 (18), 275 (24), 241 (35), 194 (72), 191 (26), 165 (60), 152 (100), 147 (26), 139 (30), 127 (35), 121 (35), 115 (21), 106 (16), 87 (12), 84 (21), 77 (15), 63 (23). Anal. Calcd (%) for $C_{21}H_{14}Cl_3NO_2$: C, 60.24; H, 3.37; N, 3.35. Found (%): C, 60.12; H, 3.20; N, 3.22.

(2-(3,3-Dimethyl-3*H*-benzo[*g*]indolin-2-yl)-5,6,7- trichloro-1,3-tropolone (**7b**).

A solution of 2,3,3-trimethyl-3*H*-benzo[*g*]indoline (**2b**, 1.05 g, 5 mmol) and *o*-chloranil (**3**, 1.23 g, 5 mmol) in dioxane (10 mL) was refluxed for 1.5 h. The solvent was removed *in vacuo* and the residue was dissolved in methylene chloride. The solution was purified by silica gel column chromatography with hexane/CH_2Cl_2 1:1. The orange fraction of **7b** with R_f 0.4 was collected and recrystallized from *n*-hexane. Orange crystals. Yield 28%, mp 209-210 ºC (*n*-hexane). ^{1}H NMR ($CDCl_3$) *δ* (ppm), *J*/Hz: 1.75 (s, 6H, 2Me), 6.93 (s, 1H, H^4), 7.44 (d, 1H, H_{Ar}, *J* = 8.4), 7.54 (t, 1H, H_{Ar}, *J* = 7.8), 7.63 (t, 1H, H_{Ar}, *J* = 8.4), 7.78 (d, 1H, H_{Ar}, *J* = 8.4), 7.91 (d, 1H, H_{Ar}, *J* = 8.4), 7.98 (d, 1H, H_{Ar}, *J* = 7.8), 15.76 (br. s, 1H, OH). ^{13}C NMR ($CDCl_3$) *δ* (ppm): 25.4, 53.6, 112.4, 115.1, 122.3, 124.4, 126.8, 127.2, 128.0, 128.2, 129.0, 129.9, 130.1, 130.3, 130.8, 132.0, 133.4, 134.2, 135.4, 136.9, 140.0, 177.1, 191.0, 196.4. IR (ν, cm^{-1}): 3277, 3058, 3001, 2980, 2938, 2870, 1649, 1558, 1503, 1469, 1377, 1369, 1318, 1294, 1256, 1205, 1095, 1055, 944, 895, 866, 820, 790, 763, 747, 699, 686. ESI-MS, *m/z* (rel. int.): 419 (7) [M^+], 417 (7), 391 (13), 389 (13), 376 (20), 374 (20), 341 (12), 339 (18), 304 (10), 275 (13), 240 (25), 238 (10), 220 (16), 204 (14), 194 (51), 191 (39), 178 (19), 165 (92), 156 (22), 152 (100), 149 (52), 147 (77), 139 (48), 131 (15), 127 (43), 121 (53), 119 (74), 115 (41), 107 (15), 102 (16), 86 (32), 84 (75), 77 (45), 65 (24), 63 (77), 51 (26). Anal. Calcd (%) for $C_{21}H_{14}Cl_3NO_2$: C, 60.24; H, 3.37; N, 3.35. Found (%): C, 60.10; H, 3.24; N, 3.20.

2-(1,1-Dimethyl-1*H*-benzo[*e*]indolin-2-yl)-4,5,6,7-tetrachloro-1,3-tropolone (**8a**).

A solution of 1,1,2-trimethyl-1*H*-benzo[*e*]indoline (**2a**, 1.05 g, 5 mmol) and *o*-chloranil (**3**, 2.46 g, 10 mmol) in AcOH (10 mL) was kept at 50–60 °C for 20 h. The solution was diluted with water and extracted with chloroform (2 × 50 mL). The chloroform solution was washed in a separating funnel with water (3 × 50 mL) and then dried for 2 h with anhydrous Na_2SO_4. The solvent was removed *in vacuo*, the residue was purified by silica gel column chromatography with hexane/CH_2Cl_2 1:1. The yellow fraction of **8a** with R_f 0.9 was collected and recrystallized from *n*-hexane. Bright yellow crystals. Yield 26%, mp 226-227 ºC (*n*-hexane). ^{1}H NMR ($CDCl_3$) δ (ppm), *J*/Hz: 1.99 (s, 6H, 2Me), 7.42 (d, 1H, H_{Ar}, *J* = 8.4), 7.53 (t, 1H, H_{Ar}, *J* = 7.8), 7.65 (t, 1H, H_{Ar}, *J* = 8.4), 7.91 (d, 1H, H_{Ar}, *J* = 8.4), 7.97 (d, 1H, H_{Ar}, *J* = 8.4), 8.08 (d, 1H, H_{Ar}, *J* = 8.6), 14.29 (br. s, 1H, OH). ^{13}C NMR ($CDCl_3$) δ (ppm): 14.1, 22.6, 23.1, 54.5, 112.1, 112.5, 122.4, 125.4, 127.7, 127.9, 130.2, 130.5, 132.7, 134.7, 135.6, 179.0. IR (v, cm^{-1}): 3128, 3058, 2973, 2929, 2869, 1666, 1626, 1599, 1561, 1524, 1495, 1471, 1443, 1380, 1366, 1345, 1246, 1213, 1129, 1105, 1067, 962, 940, 924, 867, 813, 803, 773, 752, 740, 696. ESI-MS, *m/z* (rel. int.): 453 (80) [M+], 424 (27), 416 (17), 402 (100), 389 (22), 380 (17), 374 (21), 338 (22), 309 (15), 275 (30), 238 (23), 220 (17), 194 (69), 190 (29), 165 (55), 155 (27), 152 (63), 139 (22), 126 (25), 120 (23), 118 (17), 115 (17), 111 (12), 106 (12), 87 (10), 63 (12). Anal. Calcd (%) for $C_{21}H_{13}Cl_4NO_2$: C, 55.66; H, 2.89; N, 3.09. Found (%): C, 55.44; H, 2.66; N, 2.90.

(2-(3,3-Dimethyl-3*H*-benzo[*g*]indolin-2-yl)-4,5,6,7-tetrachloro-1,3-tropolone (**8b**).

A solution of 2,3,3-trimethyl-3*H*-benzo[*g*]indoline (**2b**, 1.05 g, 5 mmol) and *o*-chloranil (**3**, 2.46 g, 10 mmol) in AcOH (10 mL) was kept at 60 °C for 72 h. The solution was diluted with water and extracted with chloroform (2 × 50 mL). The chloroform solution was washed in a separating funnel with water (3 × 50 mL) and then dried for 2 h with anhydrous Na_2SO_4. The solvent was removed *in vacuo*, the residue was purified by silica gel column chromatography with hexane/CH_2Cl_2 1:1. The yellow-orange fraction of **8b** with R_f 0.8 was collected and recrystallized from *n*-hexane. Bright yellow crystals. Yield 24%, mp 152-153 ºC (*n*-hexane). IR (ν, cm^{-1}): 3312, 3007, 2979, 2933, 2872, 1665, 1578, 1561, 1544, 1495, 1466, 1445, 1396, 1370, 1322, 1279, 1249, 1219, 1207, 1071, 1037, 980, 898, 869, 823, 798 ,777, 756, 688, 675. ^{1}H NMR ($CDCl_3$) *δ* (ppm), *J*/Hz: 1.75 (s, 6H, 2Me), 7.44 (d, 1H, H_{Ar}, *J* = 7.8), 7.55 (t, 1H, H_{Ar}, *J* = 7.8), 7.63 (t, 1H, H_{Ar}, *J* = 8.4), 7.79 (d, 1H, H_{Ar}, *J* = 8.4), 7.92 (d, 1H, H_{Ar}, *J* = 8.4), 7.98 (d, 1H, H_{Ar}, *J* = 8.4), 14.84 (br. s, 1H, OH). ^{13}C NMR ($CDCl_3$) *δ* (ppm): 23.8, 53.7, 112.9, 119.2, 120.6, 120.7, 126.7, 126.9, 127.5, 128.8, 133.5, 133.7, 138.4, 177.9. ESI-MS, *m/z* (rel. int.): 453 (76) [M^+], 424 (17), 416 (14), 402 (100), 400 (98), 389 (15), 380 (16), 373 (19), 338 (27), 309 (15), 275 (36), 238 (27), 220 (27), 207 (18), 194 (85), 190 (48), 180 (21), 165 (89), 155 (42), 152 (86), 139 (38), 137 (25), 126 (41), 120 (42), 118 (29), 115 (29), 111 (22), 106 (16), 87 (17), 77 (17), 63 (24), 57 (17). Anal. Calcd (%) for $C_{21}H_{13}Cl_4NO_2$: C, 55.66; H, 2.89; N, 3.09. Found (%): C, 55.48; H, 2.70; N, 2.88.
