# Supplementary material for: Synthesis, structure, ionochromic and cytotoxic properties of new 2-(indolin-2-yl)-1,3-tropolones
Source: Beilstein J Org Chem. 2025 Feb 17;21:358–68. doi: 10.3762/bjoc.21.26 (PMC11849549; doi:10.3762/bjoc.21.26)
Supplement: File 2 — 1H, 13C NMR, IR and HRMS spectra of all novel compounds. [file Beilstein_J_Org_Chem-21-358-s002.pdf]

BEILSTEIN JOURNAL OF ORGANIC CHEMISTRY

Supporting Information

for

Synthesis, structure, ionochromic and cytotoxic properties of new 2-(indolin-2-yl)-1,3-tropolones

Yurii A. Sayapin, Eugeny A. Gusakov, Inna O. Tupaeva, Alexander D. Dubonosov, Igor V. Dorogan, Valery V. Tkachev, Anna S. Goncharova, Gennady V. Shilov, Natalia S. Kuznetsova, Svetlana Y. Filippova, Tatyana A. Krasnikova, Yanis A. Boumber, Alexey Y. Maksimov, Sergey M. Aldoshin and Vladimir I. Minkin

Beilstein J. Org. Chem. **2025,** *21,* 358–368. doi:10.3762/bjoc.21.26

^{1}H, ^{13}C NMR, IR and HRMS spectra of all novel compounds

License and Terms: This is a supporting information file under the terms of the Creative Commons Attribution License (https://creativecommons.org/licenses/by/4.0). Please note that the reuse, redistribution and reproduction in particular requires that the author(s) and source are credited and that individual graphics may be subject to special legal provisions.
The license is subject to the *Beilstein Journal of Organic Chemistry* terms and conditions: (https://www.beilstein-journals.org/bjoc/terms)

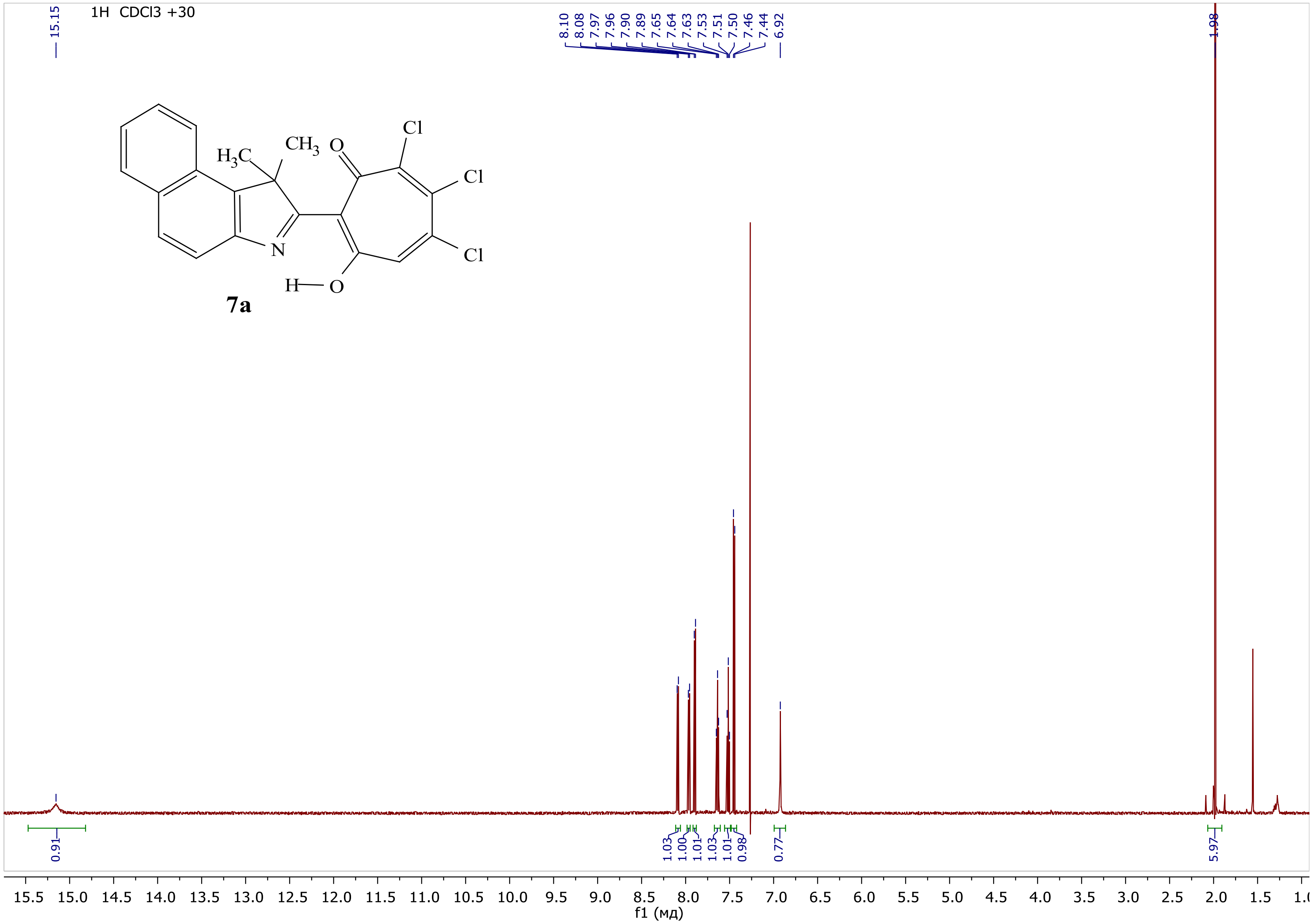

Figure S1. ^{1}H NMR spectrum of compound **7a** in $CDCl_3$.

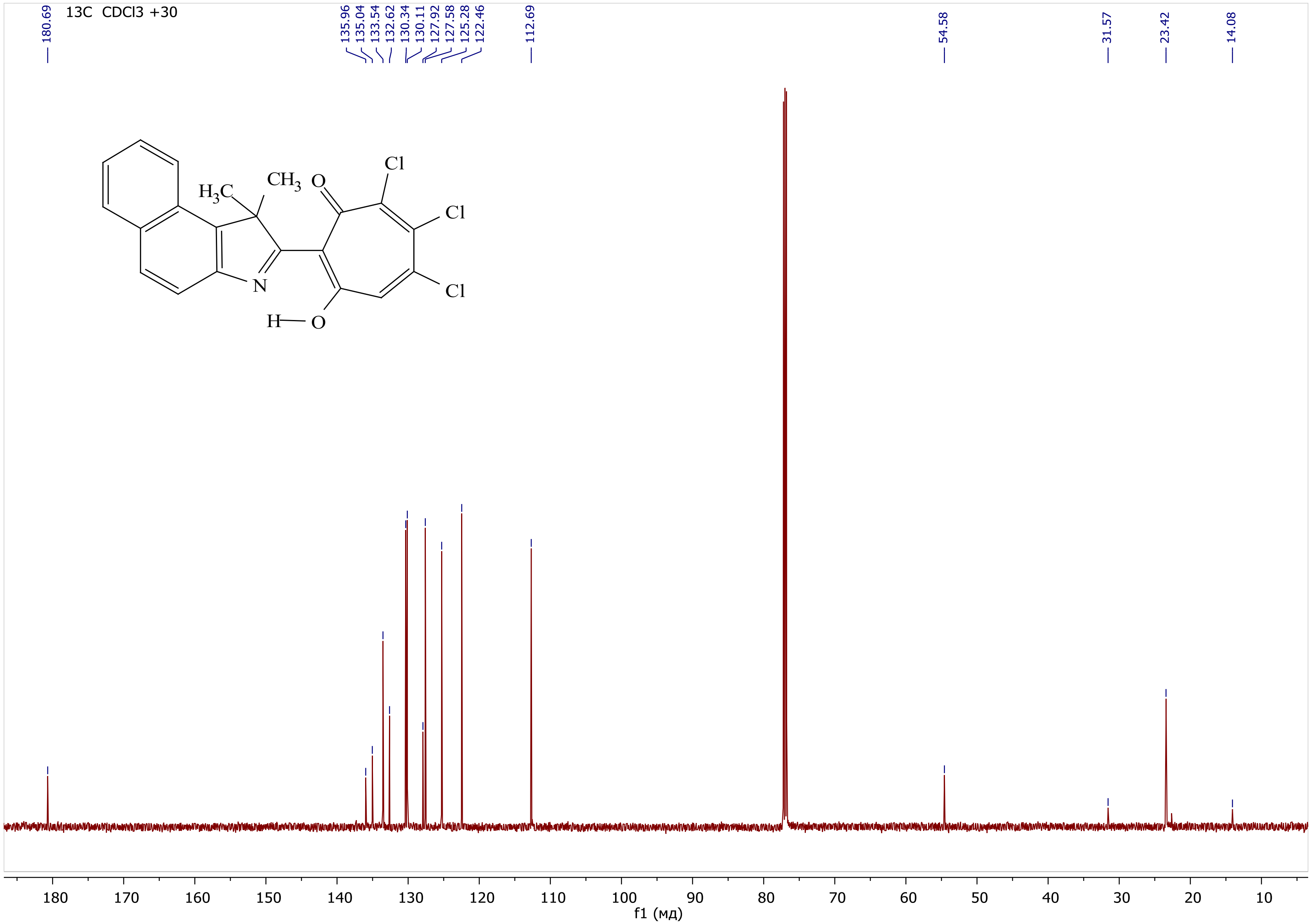

Figure S2. ^{13}C NMR spectrum of compound **7a** in $CDCl_3$.

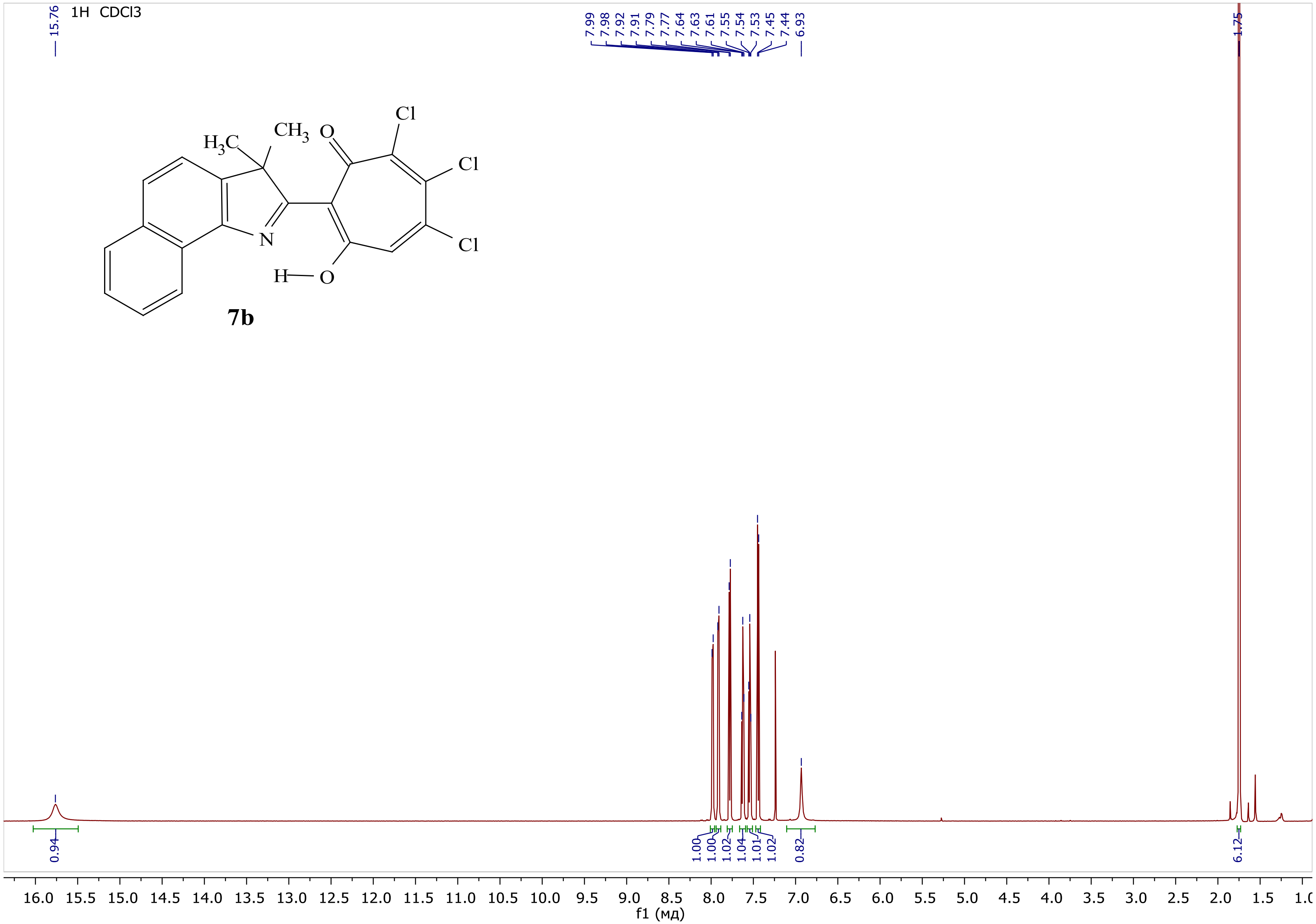

Figure S3. ^{1}H NMR spectrum of compound **7b** in $CDCl_3$.

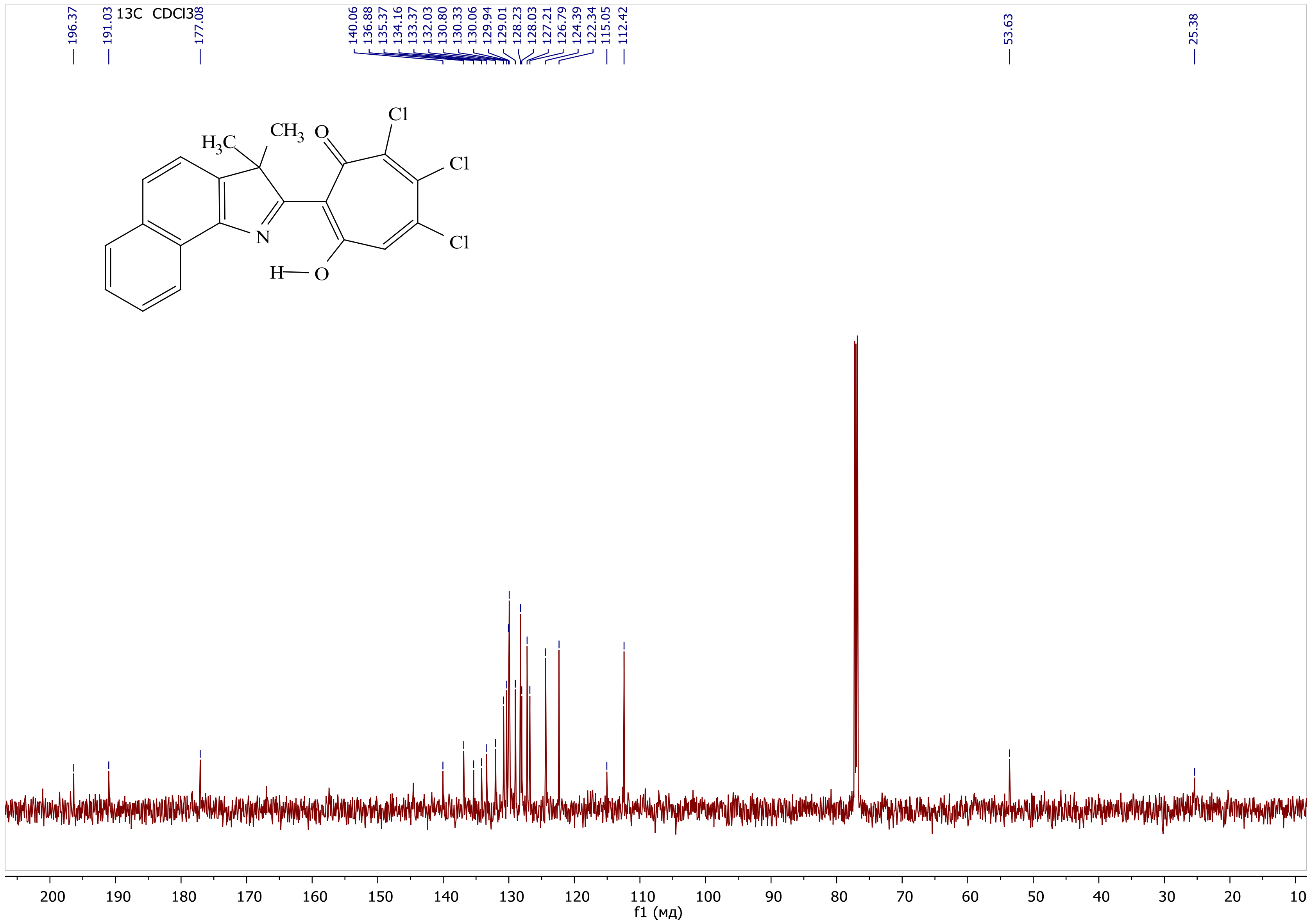

Figure S4. ^{13}C NMR spectrum of compound **7b** in $CDCl_3$.

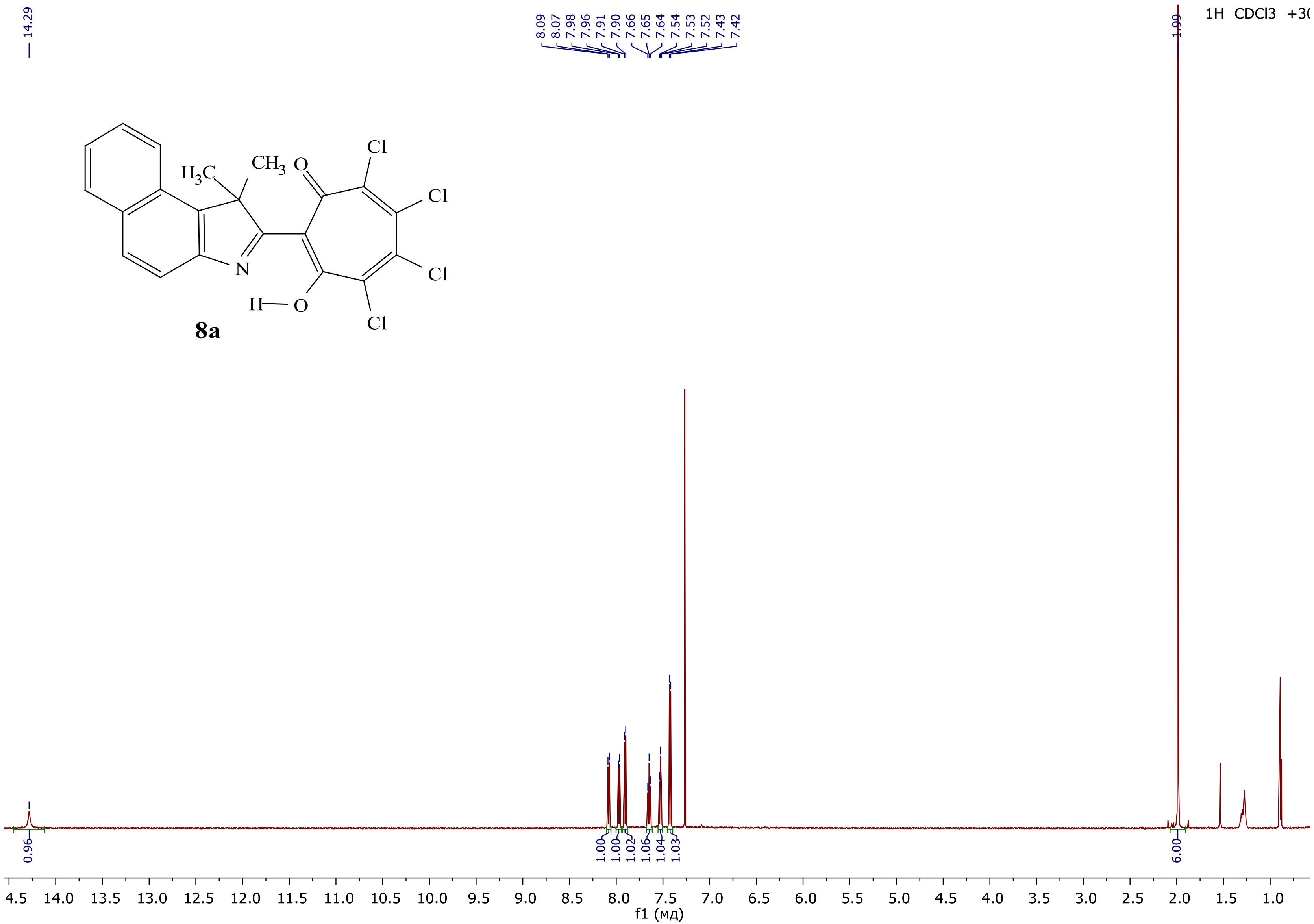

Figure S5. ^{1}H NMR spectrum of compound **8a** in $CDCl_3$.

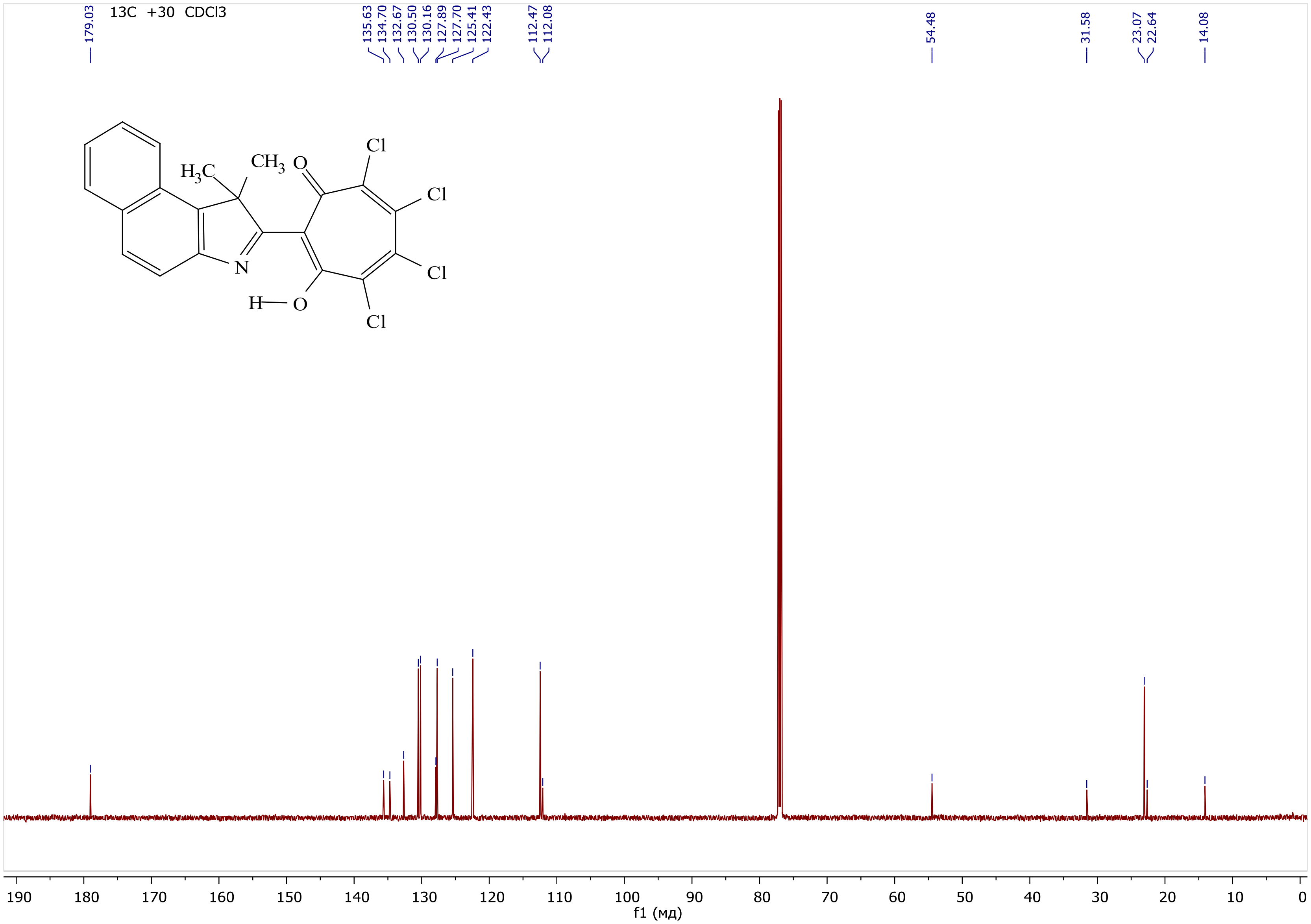

Figure S6. ^{13}C NMR spectrum of compound **8a** in $CDCl_3$.

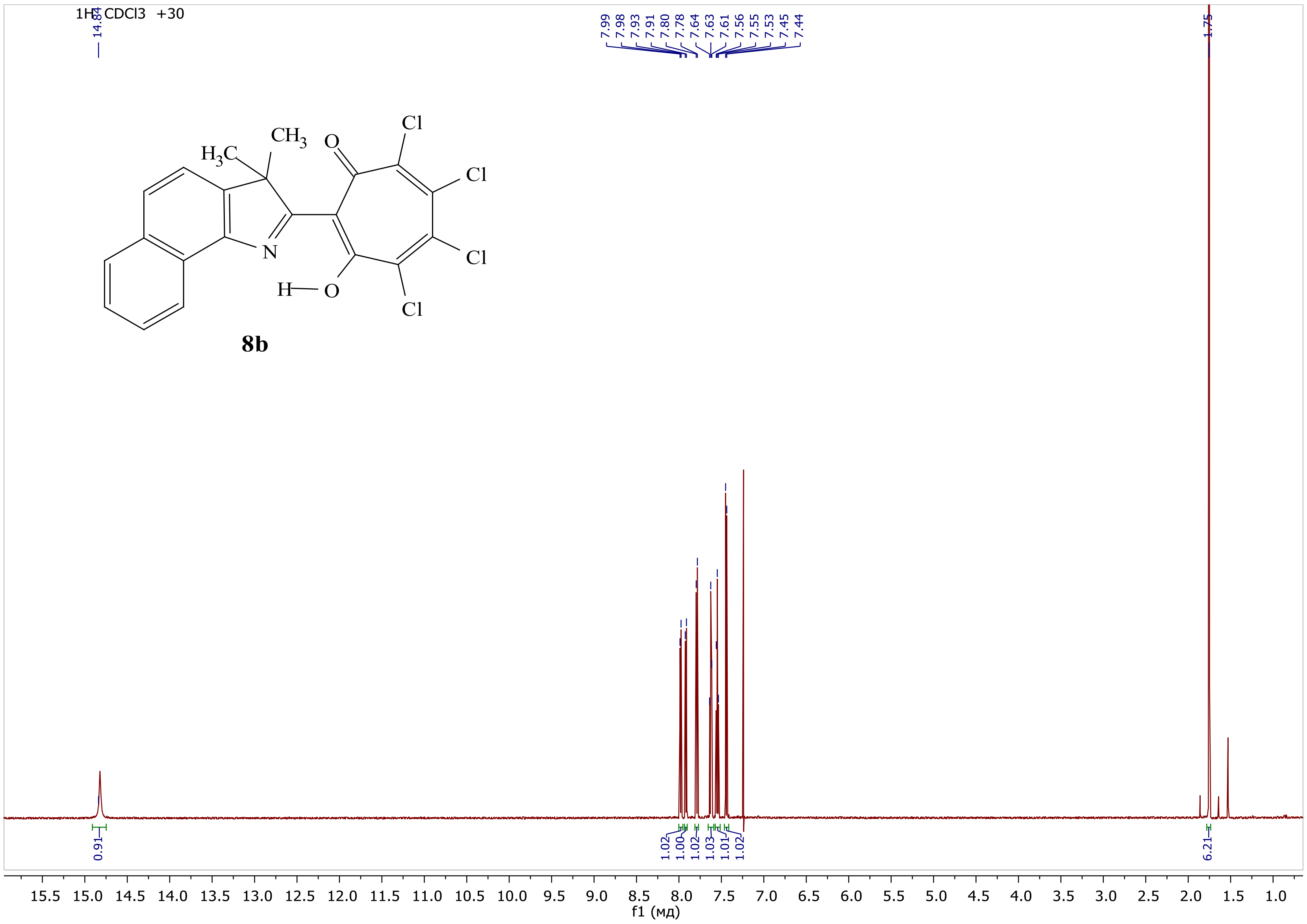

Figure S7. ^{1}H NMR spectrum of compound **8b** in $CDCl_3$.

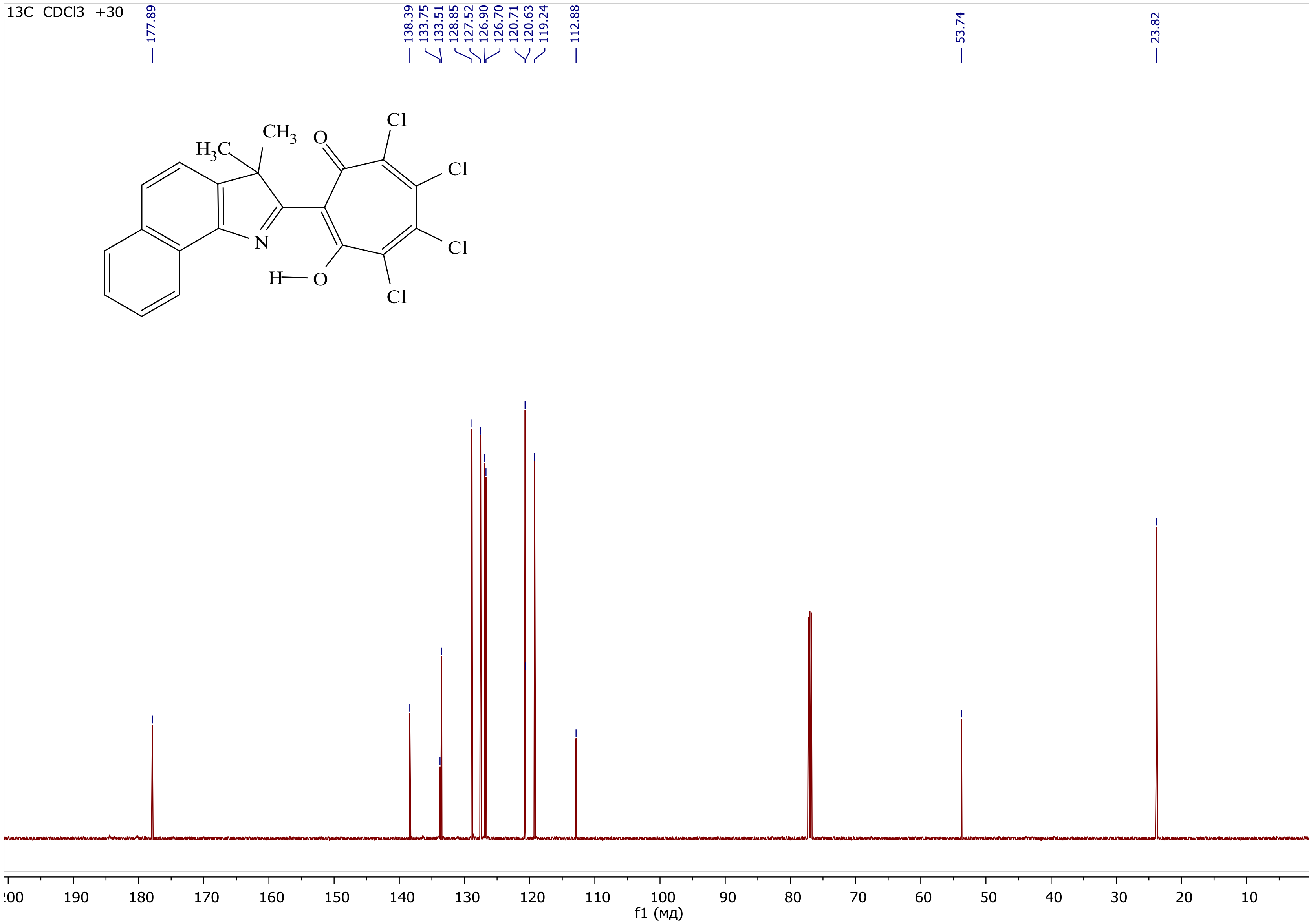

Figure S8. ^{13}C NMR spectrum of compound **8b** in $CDCl_3$.

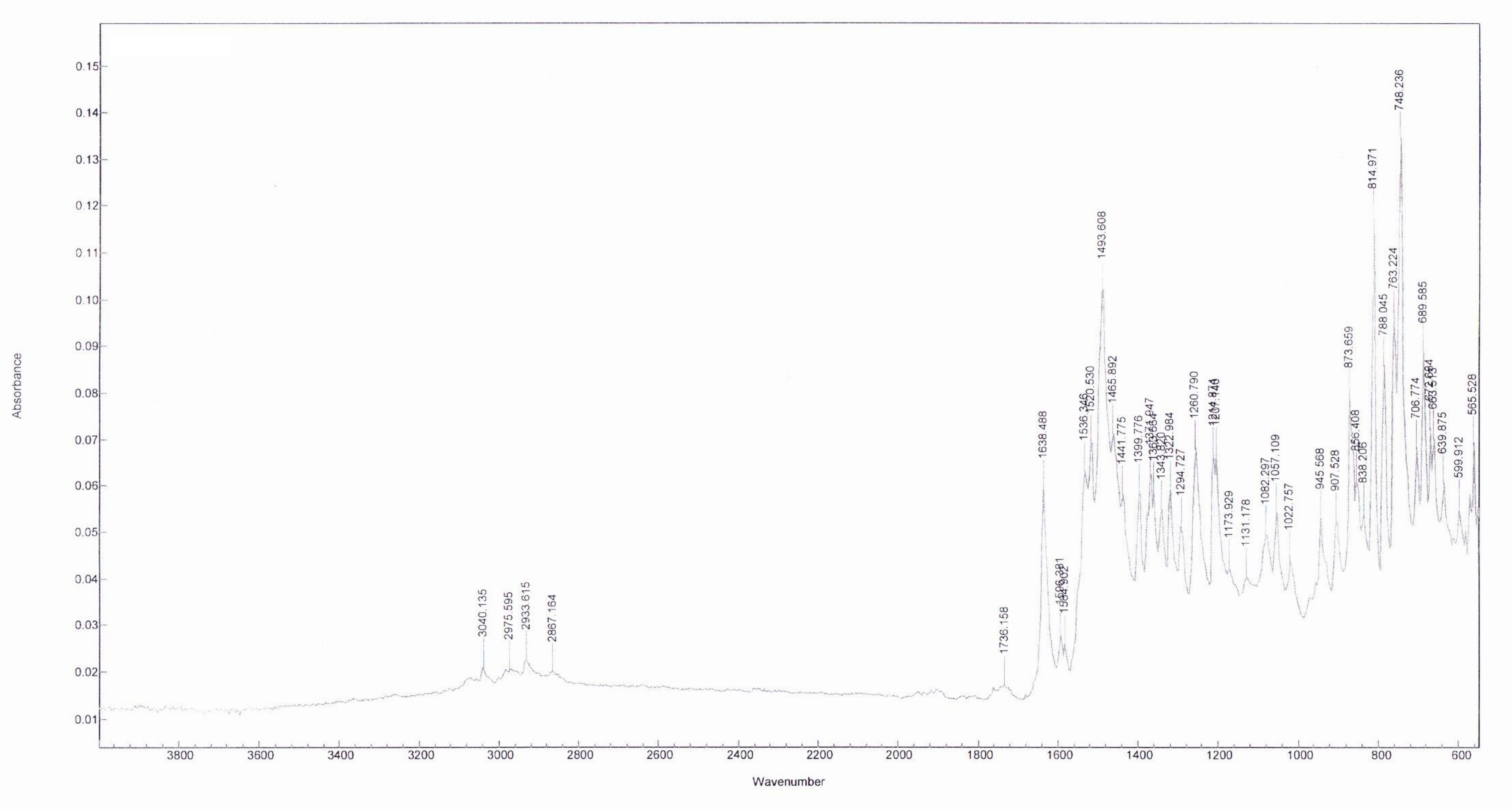

Figure S9. IR spectrum of compound **7a**.

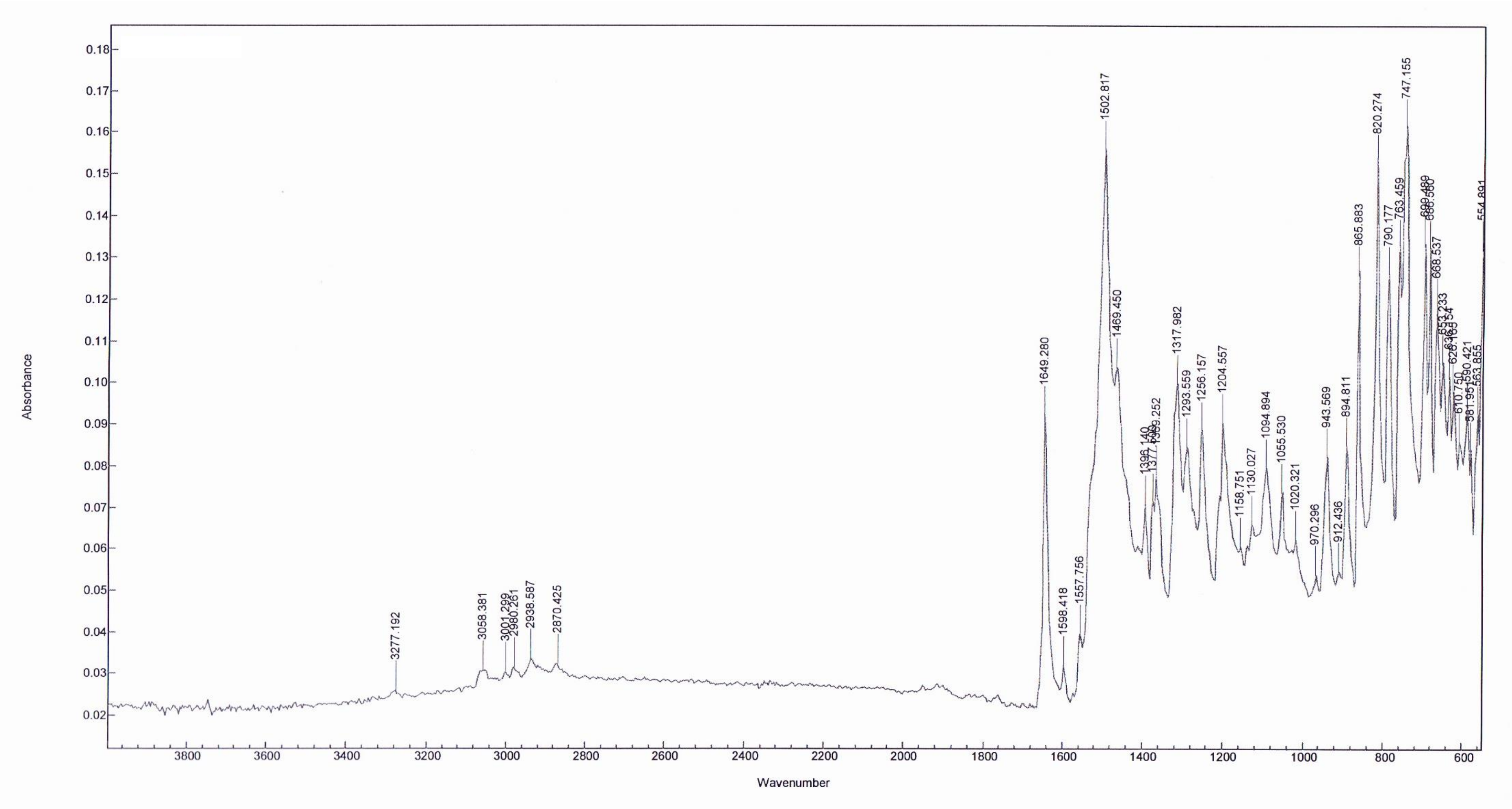

Figure S10. IR spectrum of compound **7b**.

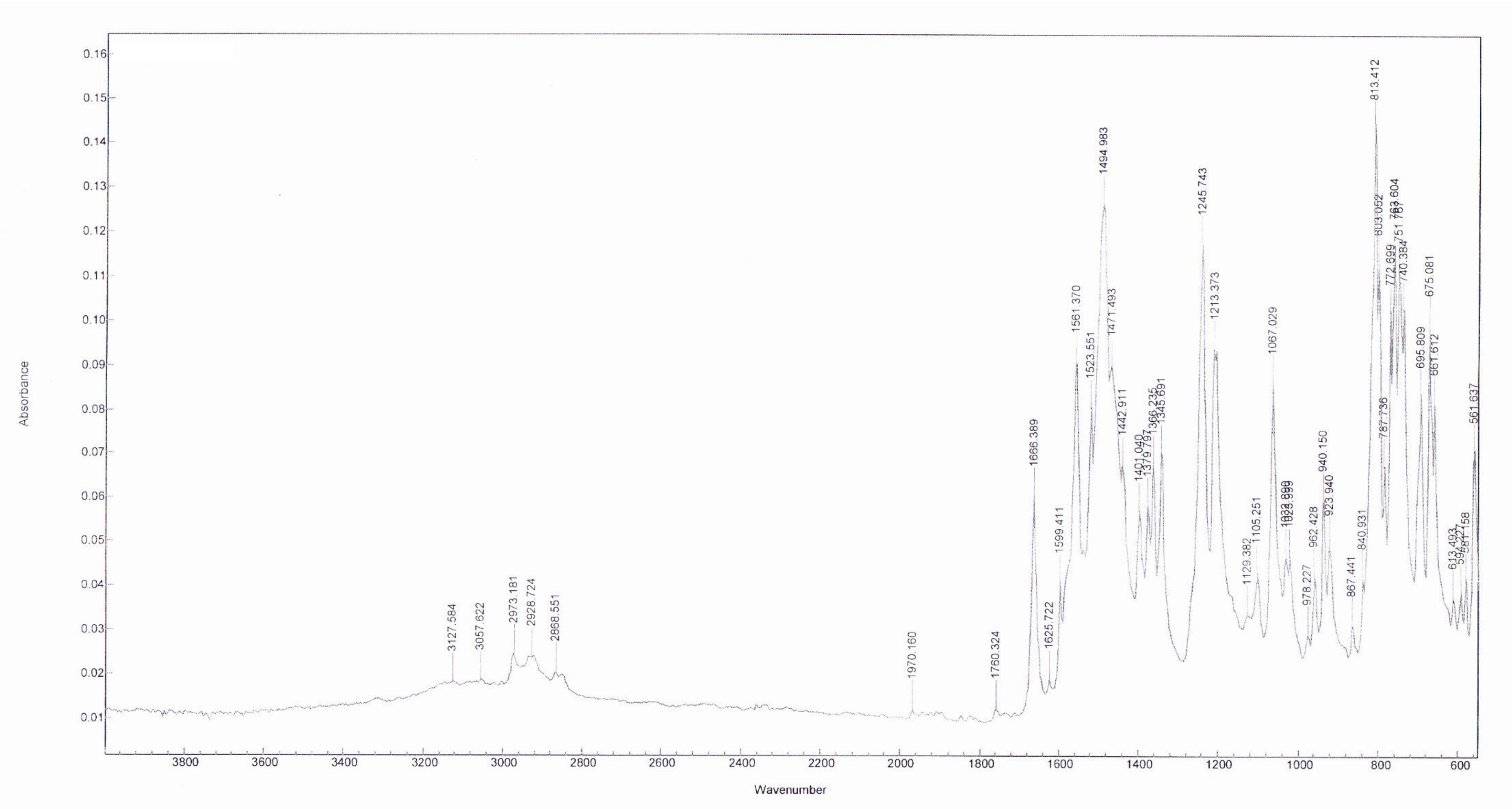

Figure S11. IR spectrum of compound **8a**.

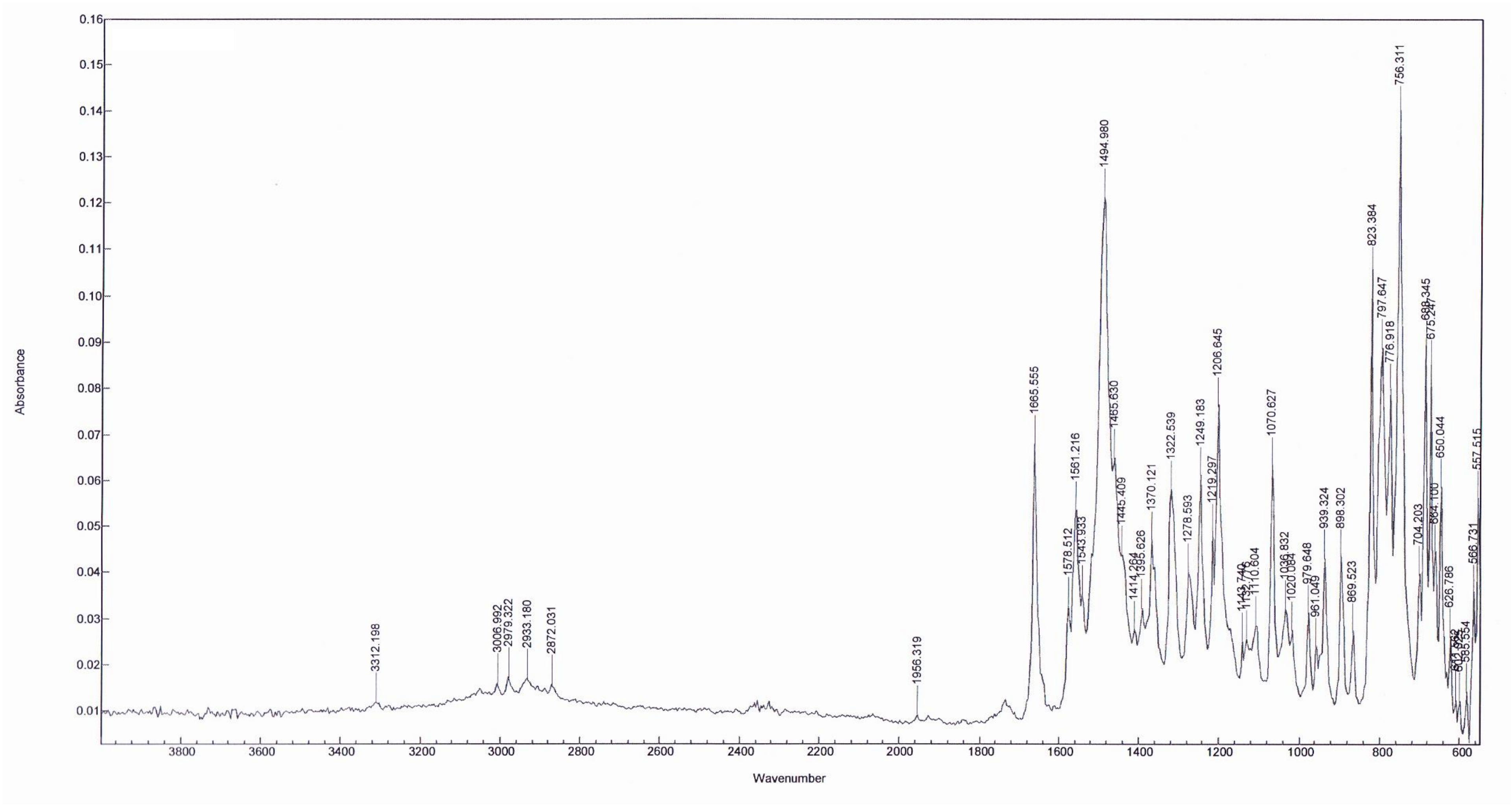

Figure S12. IR spectrum of compound **8b**.

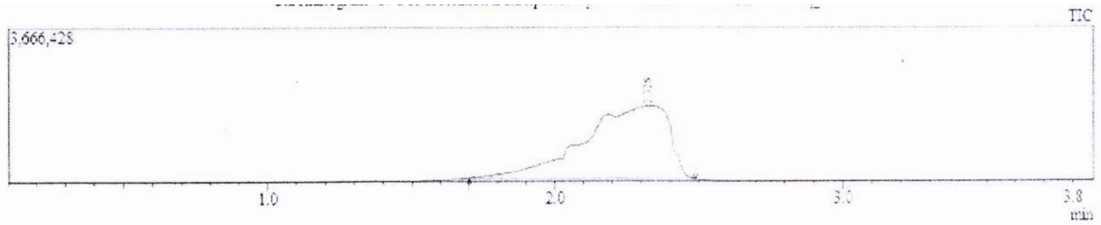

Peak Report TIC

Peak#	R.Time	Area	Area%	Base m/z	Base Int.	Name
1	2.326	36125890	100.00	373.95	61444	
		36125890	100.00			

Spectrum

Line#:1 R.Time:2.325(Scan#:446)
MassPeaks:323
RawMode:Averaged 1.710-2.470(323-475) BasePeak:152(28651)
BG Mode:None Group 1 - Event 1

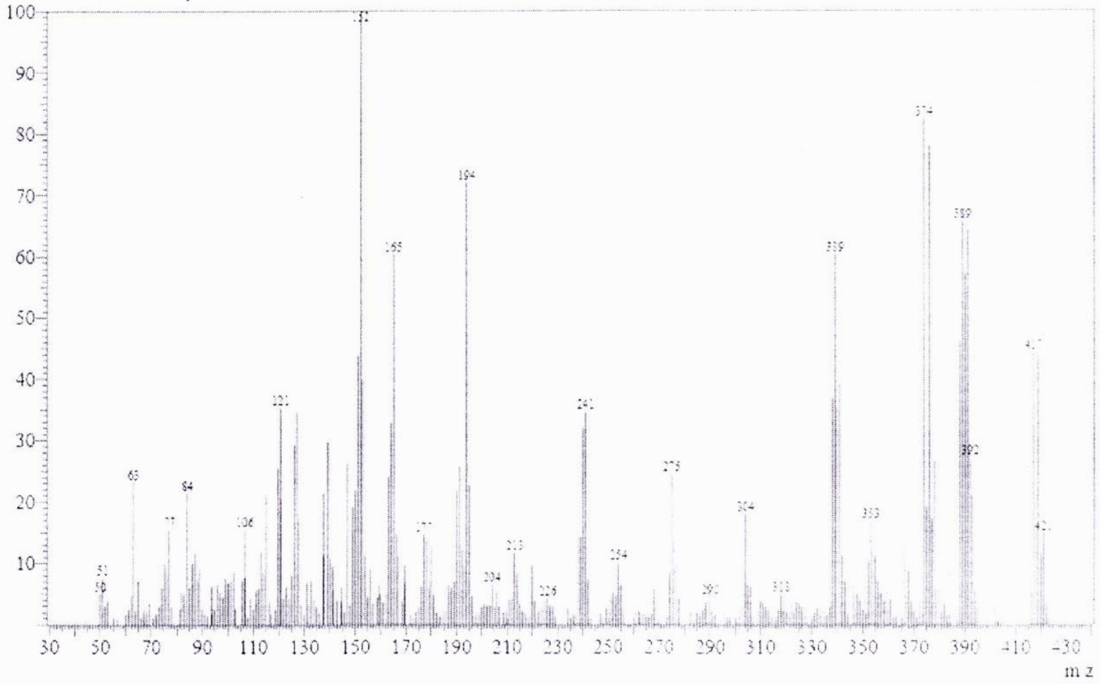

Mass Table
Line#:1 R.Time:2.325(Scan#:446)
MassPeaks:323
RawMode:Averaged 1.710-2.470(323-475) BasePeak:152(28651)
BG Mode:None Group 1 - Event 1

#	m/z	Abs. Int.	Rel. Int.
1	50.00	1399	4.88
2	51.00	2158	7.53
3	52.00	868	3.03
4	53.00	1114	3.89
5	55.10	339	1.18
6	57.00	292	1.02
7	60.10	485	1.69
8	61.05	697	2.43
9	62.00	1380	4.82
10	63.00	6664	23.26
11	64.05	629	2.20
12	65.00	2019	7.05
13	66.00	340	1.19

Figure S13. Mass spectrum of compound **7a**.

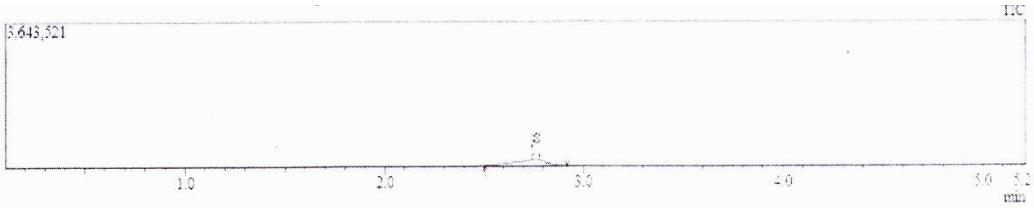

Peak Report TIC

Peak#	R.Time	Area	Area%	Base m/z	Base Int.	Name
1	2.766	1802337	100.00	152.10	6240	
		1802337	100.00			

Spectrum

Line#:1 R.Time:2.765(Scan#:534)
MassPeaks:269
RawMode:Averaged 2.520-2.910(485-563) BasePeak:152(2970)
BG Mode:None Group 1 - Event 1

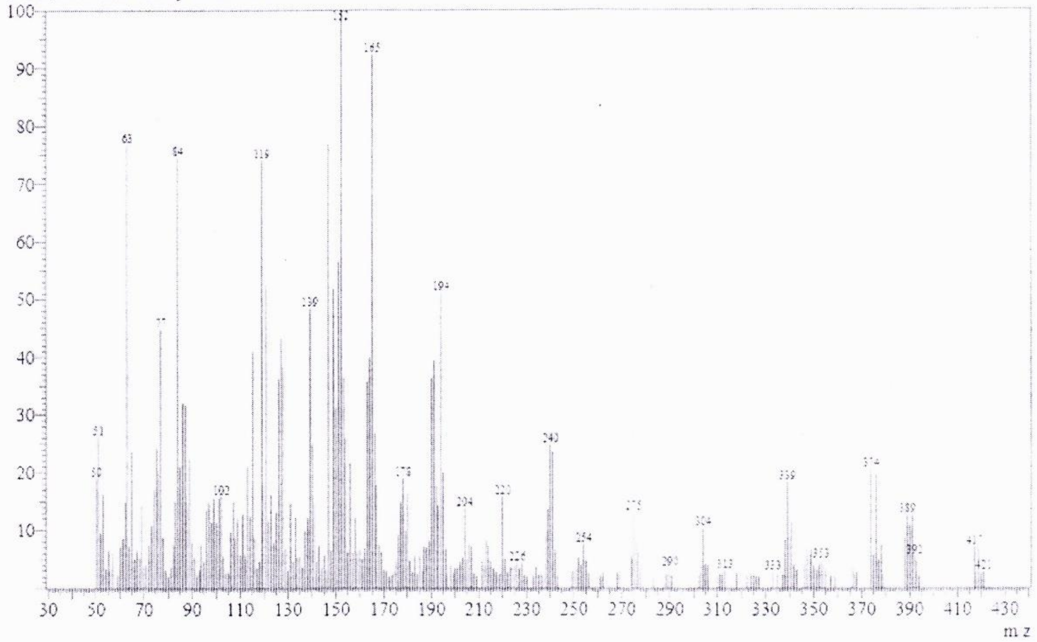

Mass Table
Line#:1 R.Time:2.765(Scan#:534)
MassPeaks:269
RawMode:Averaged 2.520-2.910(485-563) BasePeak:152(2970)
BG Mode:None Group 1 - Event 1

#	m/z	Abs. Int.	Rel. Int.
1	50.10	563	18.96
2	51.10	776	26.13
3	52.00	282	9.49
4	53.05	487	16.40
5	54.00	97	3.27
6	55.10	197	6.63
7	56.00	96	3.23
8	57.10	182	6.13
9	59.10	65	2.19
10	60.10	212	7.14
11	61.10	257	8.65
12	62.00	440	14.81
13	63.00	2275	76.60

Figure S14. Mass spectrum of compound **7b**.

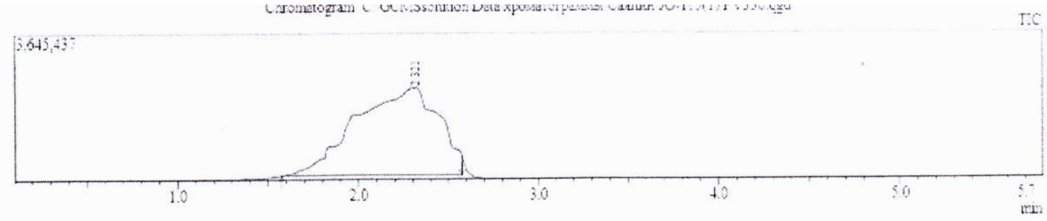

Peak Report TIC

Peak#	R.Time	Area	Area%	Base m/z	Base Int.	Name
1	2.321	70214414	100.00	401.95	76201	
		70214414	100.00			

Spectrum

Line#:1 R.Time:2.320(Scan#:445)
MassPeaks:360
RawMode:Averaged 1.610-2.560(303-493) BasePeak:402(44843)
BG Mode:None Group 1 - Event 1

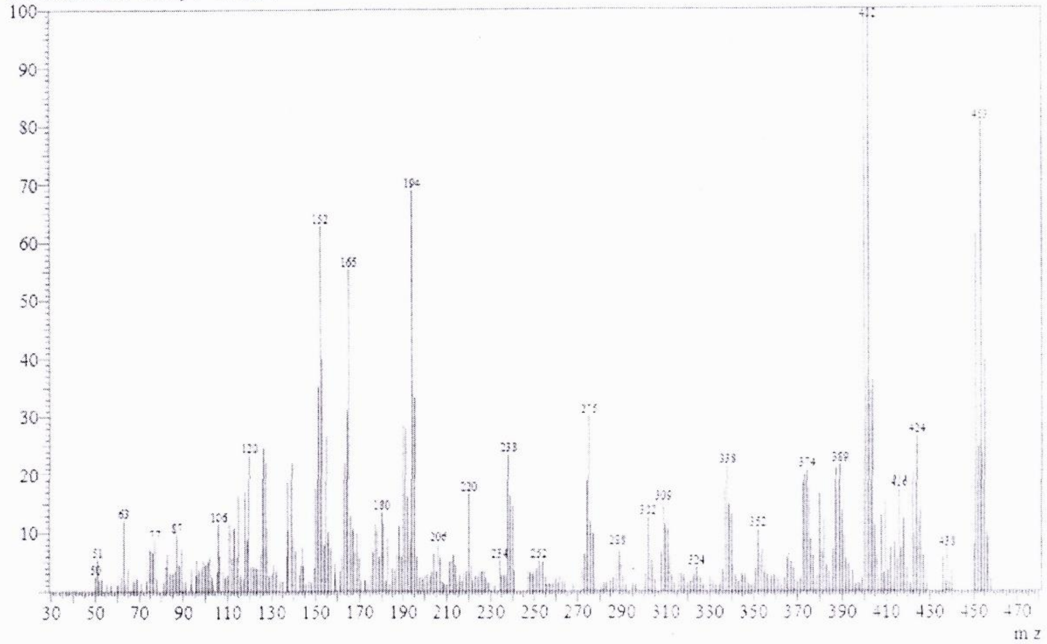

Mass Table
Line#:1 R.Time:2.320(Scan#:445)
MassPeaks:360
RawMode:Averaged 1.610-2.560(303-493) BasePeak:402(44843)
BG Mode:None Group 1 - Event 1

#	m/z	Abs. Int.	Rel. Int.
1	50.05	1148	2.56
2	51.00	2408	5.37
3	52.00	935	2.09
4	53.05	926	2.06
5	55.10	598	1.33
6	57.15	521	1.16
7	60.10	520	1.16
8	61.00	585	1.30
9	62.05	1113	2.48
10	63.00	5427	12.10
11	64.00	680	1.52
12	65.00	1732	3.86
13	67.05	825	1.84

Figure S15. Mass spectrum of compound **8a**.

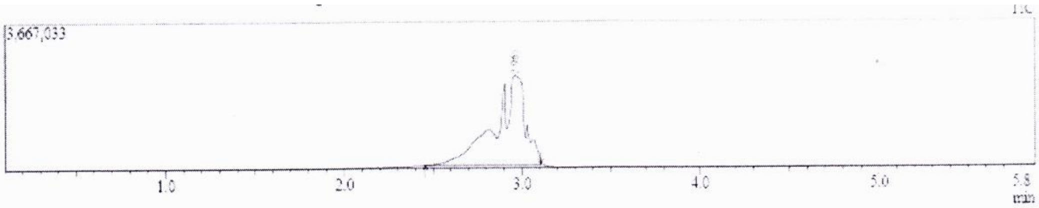

Peak Report TIC

Peak#	R.Time	Area	Area%	Base m/z	Base Int.	Name
1	2.965	26483558	100.00	402.00	86719	
		26483558	100.00			

Spectrum

Line#:1 R.Time:2.965(Scan#:574)
MassPeaks:396
RawMode:Averaged 2.475-3.080(476-597) BasePeak:402(20782)
BG Mode:None Group 1 - Event 1

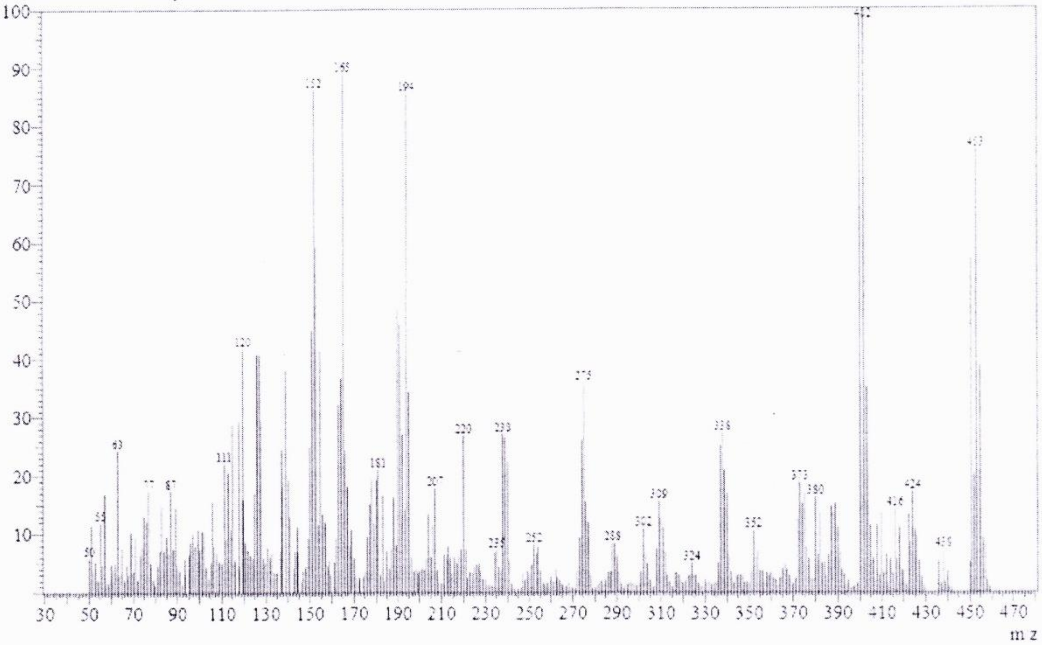

Mass Table
Line#:1 R.Time:2.965(Scan#:574)
MassPeaks:396
RawMode:Averaged 2.475-3.080(476-597) BasePeak:402(20782)
BG Mode:None Group 1 - Event 1

#	m/z	Abs. Int.	Rel. Int.
1	50.05	1194	5.75
2	51.00	2389	11.50
3	52.05	772	3.71
4	53.05	1082	5.21
5	54.10	415	2.00
6	55.10	2478	11.92
7	56.10	1001	4.82
8	57.10	3510	16.89
9	58.00	376	1.81
10	59.00	320	1.54
11	60.05	1000	4.81
12	61.00	818	3.94
13	62.00	1048	5.04

Figure S16. Mass spectrum of compound **8b**.

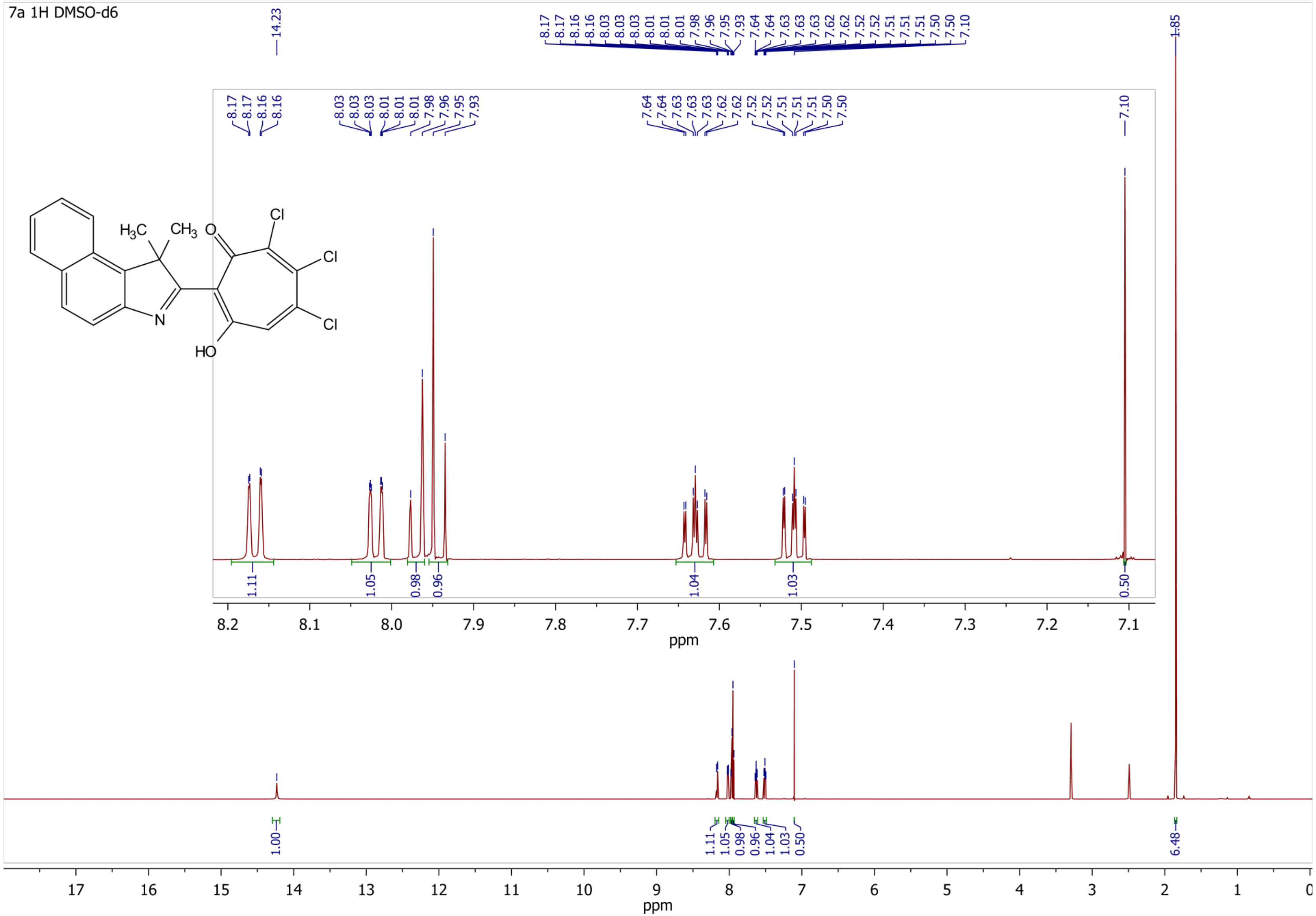

Figure S17. ^{1}H NMR spectrum of compound **7a** in DMSO-d_6.

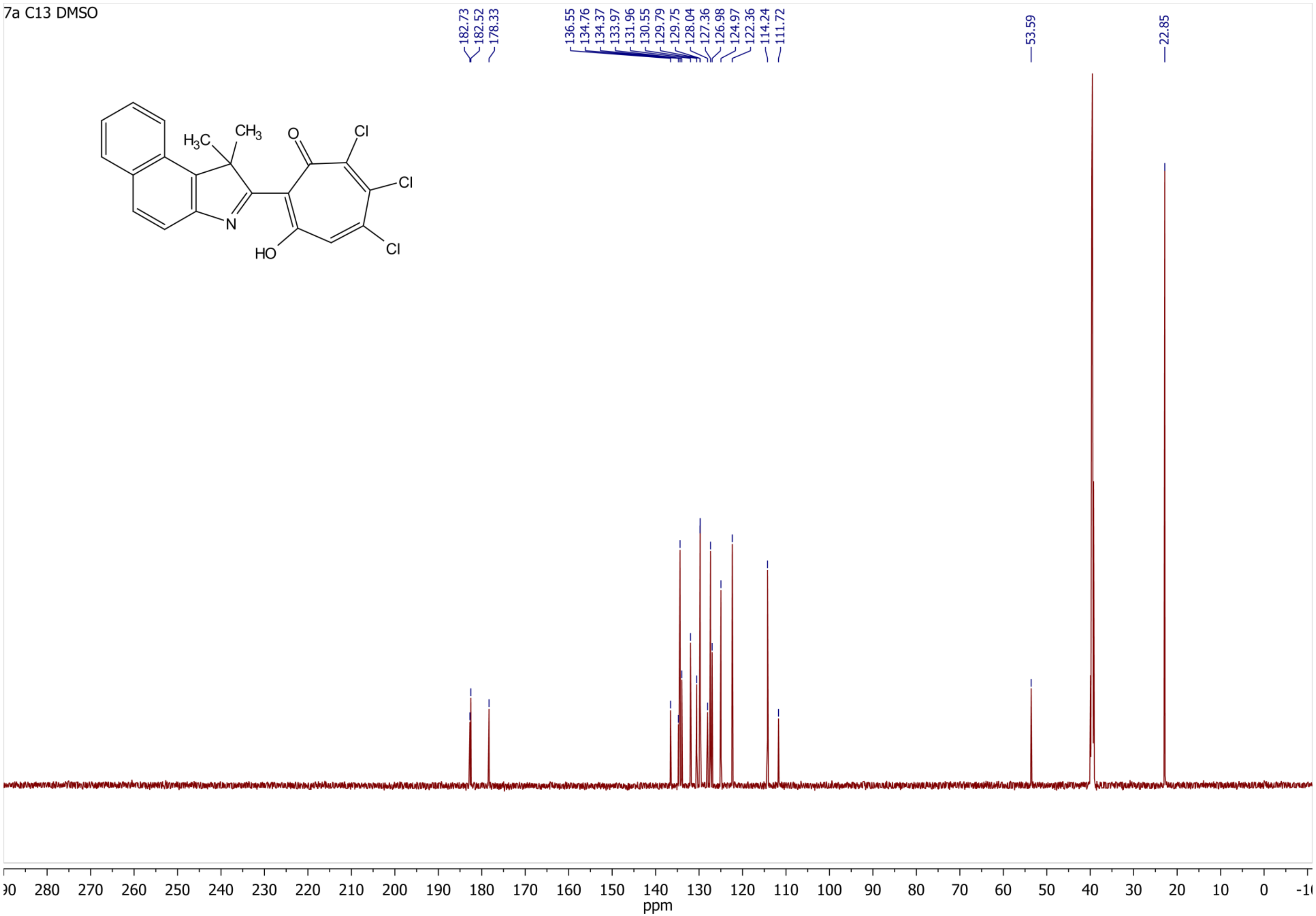

Figure S18. ^{13}C NMR spectrum of compound **7a** in DMSO-d_6.

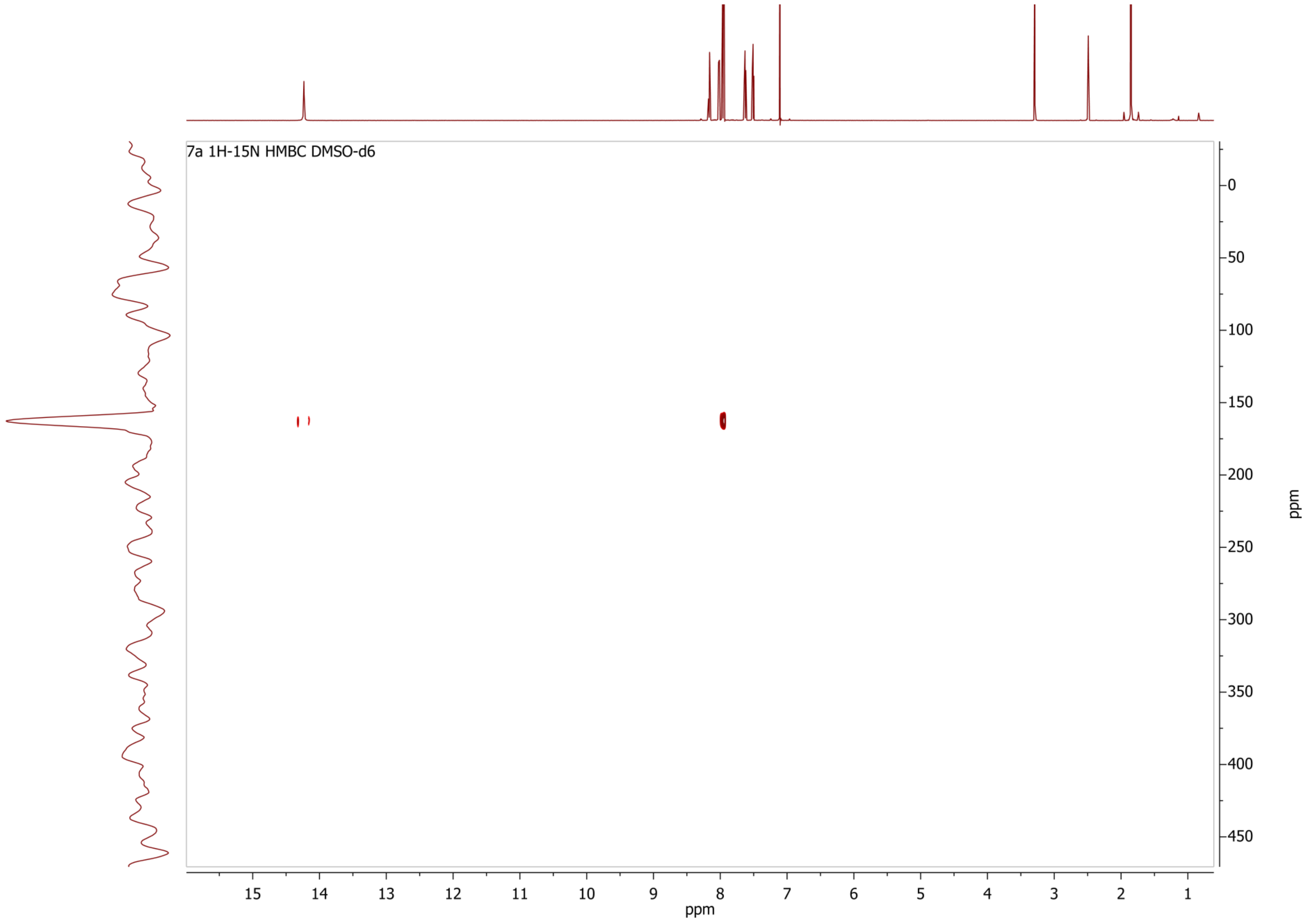

Figure S19. ^{1}H-^{15}N HMBC spectrum of compound **7a** in DMSO-d_6.

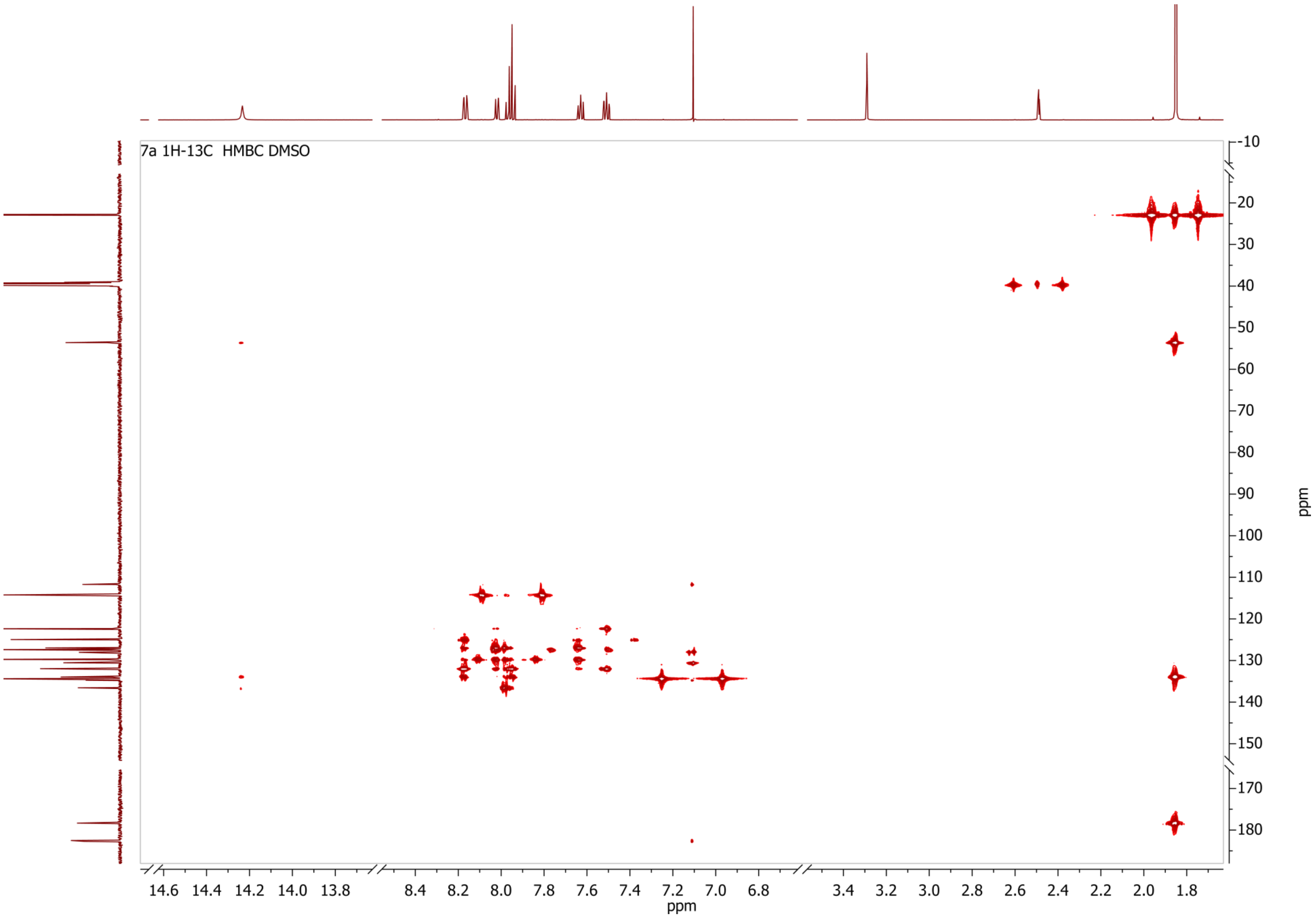

Figure S20. ^{1}H-^{13}C HMBC spectrum of compound **7a** in DMSO-d_6.

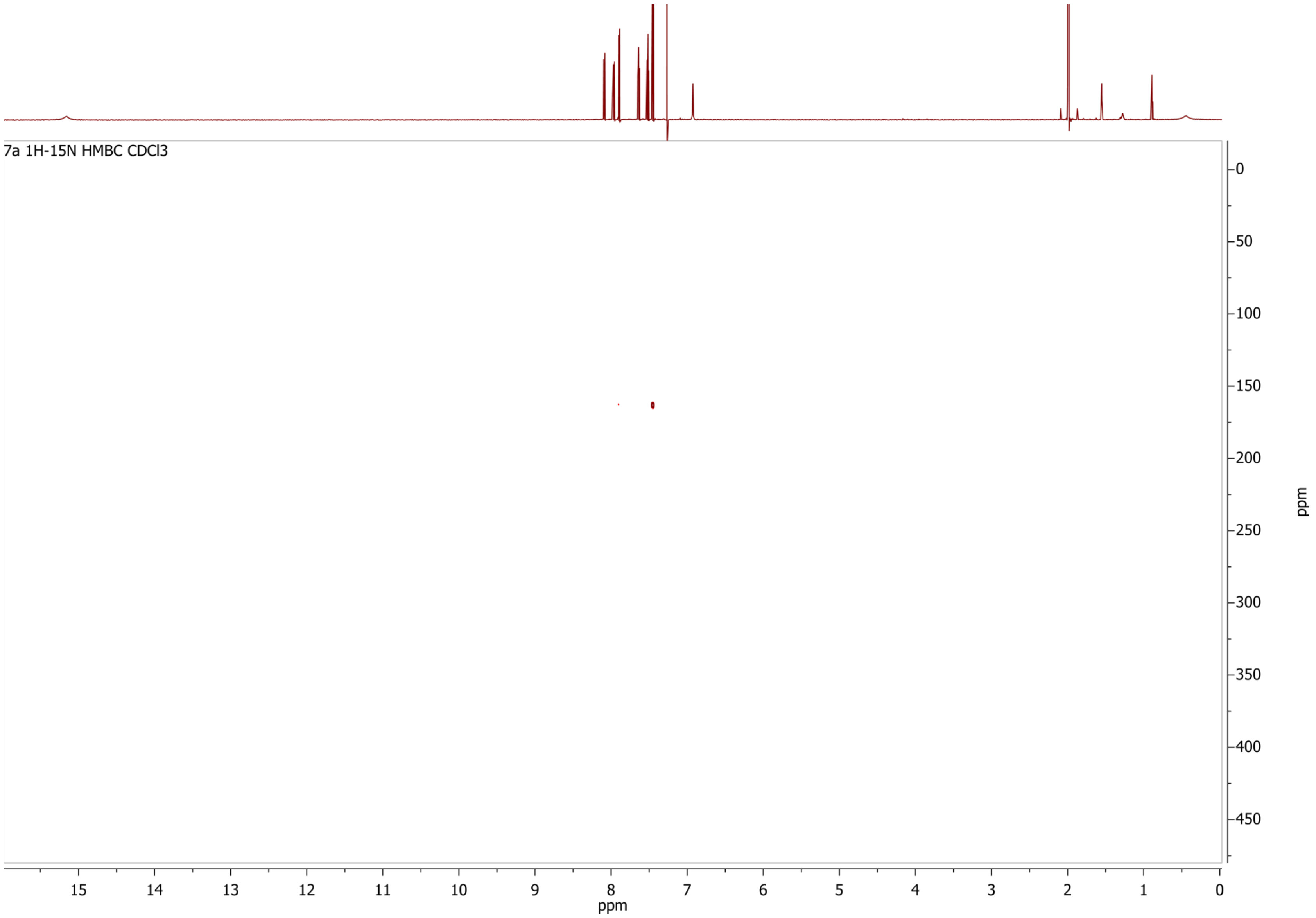

Figure S21. ^{1}H-^{15}N HMBC spectrum of compound **7a** in $CDCl_3$.

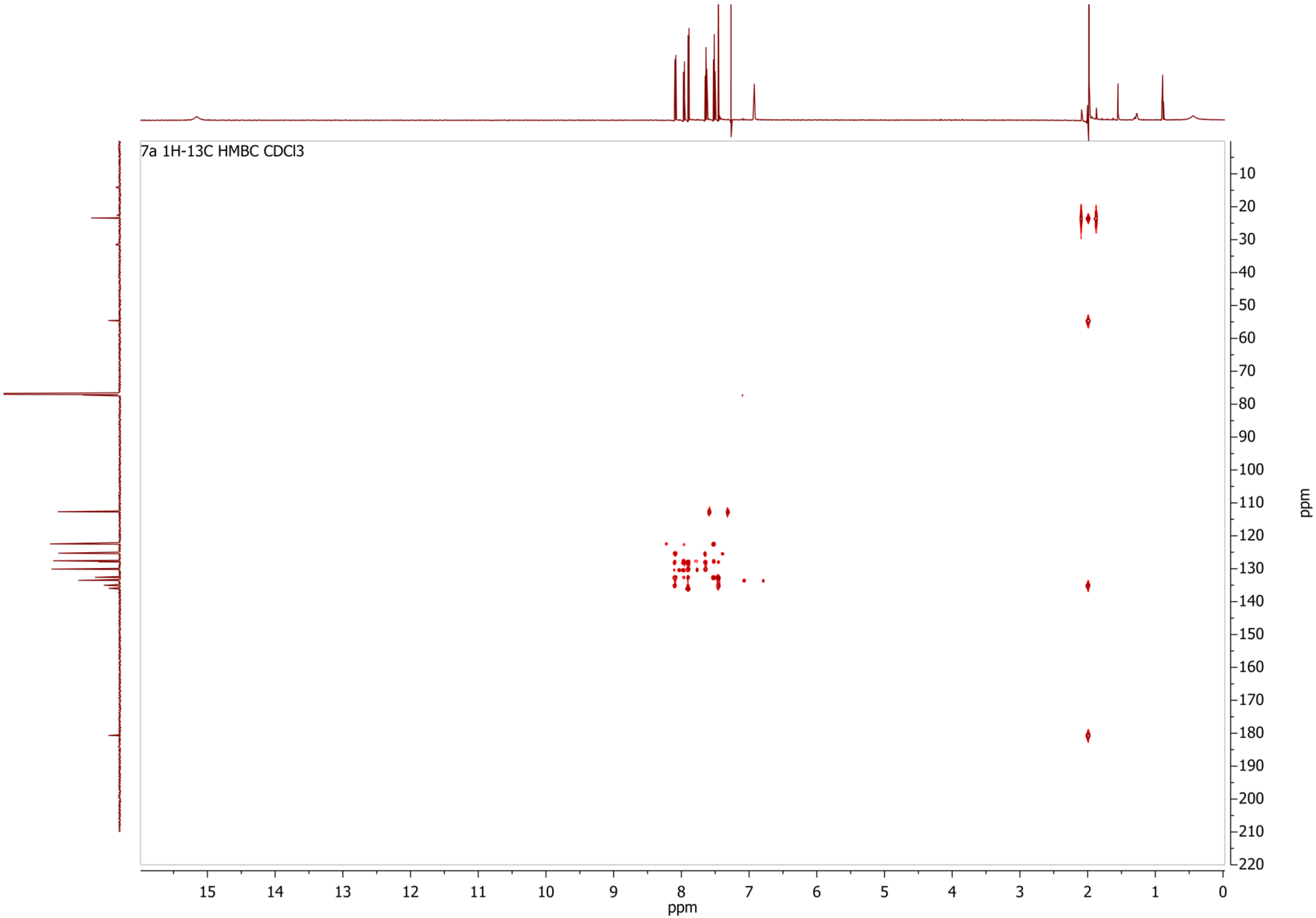

Figure S22. ^{1}H-^{13}C HMBC spectrum of compound **7a** in $CDCl_3$.
