# Supplementary material for: Synthesis, structure, ionochromic and cytotoxic properties of new 2-(indolin-2-yl)-1,3-tropolones
Source: Beilstein J Org Chem. 2025 Feb 17;21:358–68. doi: 10.3762/bjoc.21.26 (PMC11849549; doi:10.3762/bjoc.21.26)
Supplement: File 3 — X-ray analysis data of 7b and DFT quantum chemical calculations for 7a, 7b, 8a and 8b. [file Beilstein_J_Org_Chem-21-358-s003.pdf]

BEILSTEIN JOURNAL OF ORGANIC CHEMISTRY

Supporting Information

for

Synthesis, structure, ionochromic and cytotoxic properties of new 2-(indolin-2-yl)-1,3-tropolones

Yurii A. Sayapin, Eugeny A. Gusakov, Inna O. Tupaeva, Alexander D. Dubonosov, Igor V. Dorogan, Valery V. Tkachev, Anna S. Goncharova, Gennady V. Shilov, Natalia S. Kuznetsova, Svetlana Y. Filippova, Tatyana A. Krasnikova, Yanis A. Boumber, Alexey Y. Maksimov, Sergey M. Aldoshin and Vladimir I. Minkin

Beilstein J. Org. Chem. **2025,** *21,* 358–368. doi:10.3762/bjoc.21.26

X-ray analysis data of 7b and DFT quantum chemical calculations for 7a, 7b, 8a and 8b

License and Terms: This is a supporting information file under the terms of the Creative Commons Attribution License (https://creativecommons.org/licenses/by/4.0). Please note that the reuse, redistribution and reproduction in particular requires that the author(s) and source are credited and that individual graphics may be subject to special legal provisions.
The license is subject to the *Beilstein Journal of Organic Chemistry* terms and conditions: (https://www.beilstein-journals.org/bjoc/terms)

Table S1: Crystal data and structure refinement for **7b**.

CCDC Number	2040907
Empirical formula	$C_{21}H_{14}Cl_3NO_2$
Formula weight	418.68
Temperature/K	150.0(1)
Crystal system	monoclinic
Space group	$P2_1/c$
a/Å	11.4480(3)
b/Å	7.4883(2)
c/Å	21.7720(6)
α/°	90.00
β/°	102.590(3)
γ/°	90.00
Volume/Å^3	1821.55(9)
Z	4
ρ_{calc} g/cm^3	1.527
μ/mm^{-1}	0.520
F(000)	856
Crystal size/mm^3	0.31 × 0.25 × 0.20
Radiation	MoK\a (λ = 0.7107)
Index ranges	-17 ≤ h ≤ 17, -11 ≤ k ≤8, -32 ≤ l ≤23
Reflections collected	14246
Independent reflections	6352[R_{int} = 0.0214, R_{sigma} = 0.0323]
Data/restraints/parameters	6352/0/247
Goodness-of-fit on F^2	1.027
Final R indexes [I>=2σ (I)]	R_1 = 0.0385, wR_2 = 0.0928
Final R indexes [all data]	R_1 = 0.0534, wR_2 = 0.1003
Largest diff. peak/hole / e Å^{-3}	0.431/-0.340

Table S2: Bond lengths *d* (Å) and angles *ω* (deg) in **7b**.

Bond	*d*/Å	Bond	*d*/Å	Bond	*d*/Å
Cl(1)-C(5)	1.7393(13)	N(1)-C(8)	1.3381(16)	C(2)-C(8)	1.4139(17)
Cl(2)-C(6)	1.7303(12)	N(1)-C(19)	1.4040(16)	C(2)-C(3)	1.4459(17)
Cl(3)-C(7)	1.7222(13)	N(1)-H(1)	0.857(18)	C(3)-C(4)	1.4783(18)
O(1)-C(1)	1.2194(16)	C(1)-C(2)	1.4702(17)	C(4)-C(5)	1.3391(18)
O(2)-C(3)	1.2543(15)	C(1)-C(7)	1.5170(17)	C(5)-C(6)	1.4636(18)
C(6)-C(7)	1.3407(18)	C(9)-C(20)	1.549(2)	C(12)-C(13)	1.420(2)
C(8)-C(9)	1.5391(18)	C(10)-C(19)	1.3722(19)	C(13)-C(14)	1.423(2)
C(9)-C(10)	1.5156(19)	C(10)-C(11)	1.4064(19)	C(13)-C(18)	1.431(2)
C(9)-C(21)	1.5316(19)	C(11)-C(12)	1.372(2)	C(14)-C(15)	1.364(3)
C(15)-C(16)	1.415(2)	C(16)-C(17)	1.3754(19)	C(17)-C(18)	1.414(2)
**Angle**	***ω*/deg**	**Angle**	***ω*/deg**	**Angle**	***ω*/deg**
C(8)-N(1)-C(19)	112.37(11)	C(3)-C(2)-C(1)	120.92(11)	C(7)-C(6)-C(5)	122.86(11)
C(8)-N(1)-H(1)	115.4(11)	O(2)-C(3)-C(2)	122.75(11)	C(7)-C(6)-Cl(2)	120.01(10)
C(19)-N(1)-H(1)	131.9(11)	O(2)-C(3)-C(4)	114.20(11)	C(5)-C(6)-Cl(2)	117.09(9)
O(1)-C(1)-C(2)	124.45(11)	C(2)-C(3)-C(4)	122.84(11)	C(6)-C(7)-C(1)	127.44(11)
O(1)-C(1)-C(7)	118.05(11)	C(5)-C(4)-C(3)	129.68(12)	C(6)-C(7)-Cl(3)	120.95(10)
C(2)-C(1)-C(7)	117.00(11)	C(4)-C(5)-C(6)	126.57(11)	C(1)-C(7)-Cl(3)	111.61(9)
C(8)-C(2)-C(3)	118.81(11)	C(4)-C(5)-Cl(1)	117.19(10)	N(1)-C(8)-C(2)	120.54(11)
C(8)-C(2)-C(1)	120.26(11)	C(6)-C(5)-Cl(1)	116.22(9)	N(1)-C(8)-C(9)	108.17(11)
C(2)-C(8)-C(9)	131.14(11)	C(19)-C(10)-C(9)	109.60(11)	C(14)-C(15)-C(16)	120.71(14)
C(10)-C(9)-C(21)	111.95(12)	C(11)-C(10)-C(9)	129.96(13)	C(17)-C(16)-C(15)	120.28(15)
C(10)-C(9)-C(8)	100.67(10)	C(12)-C(11)-C(10)	118.44(15)	C(16)-C(17)-C(18)	119.94(14)
C(21)-C(9)-C(8)	114.77(11)	C(11)-C(12)-C(13)	121.75(14)	C(19)-C(18)-C(17)	124.49(13)
C(10)-C(9)-C(20)	107.26(10)	C(12)-C(13)-C(14)	121.86(14)	C(19)-C(18)-C(13)	115.27(13)
C(21)-C(9)-C(20)	111.53(12)	C(12)-C(13)-C(18)	120.38(13)	C(17)-C(18)-C(13)	120.19(12)
C(8)-C(9)-C(20)	109.96(11)	C(14)-C(13)-C(18)	117.74(15)	C(10)-C(19)-N(1)	108.59(11)
C(19)-C(10)-C(11)	120.30(13)	C(15)-C(14)-C(13)	121.04(15)	C(10)-C(19)-C(18)	123.74(12)

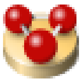

7b.cif

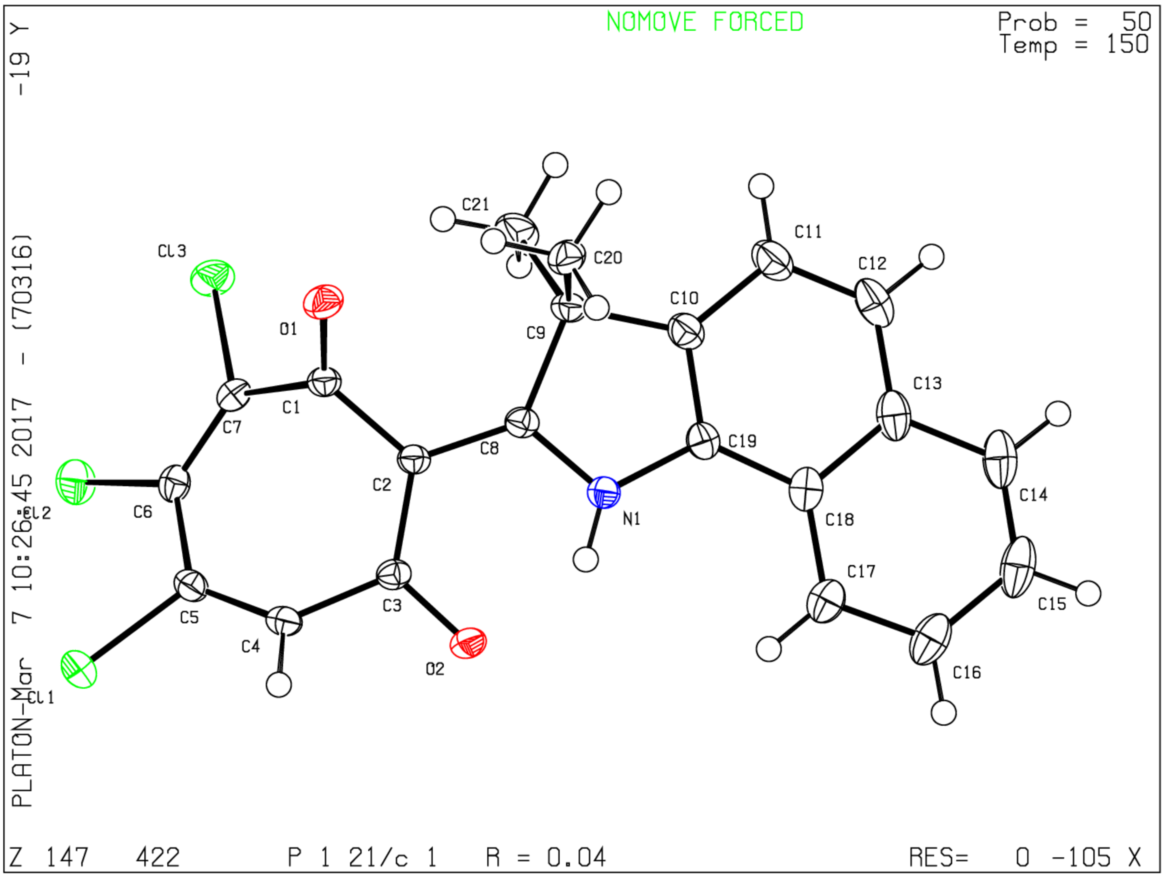
NOMOVE FORCED
Prob = 50
Temp = 150
-19 Y
PLATON-Mar 7 10:26:45 2017 - (70316)
Z 147 422
P 1 21/c 1
R = 0.04
RES= 0 -105 X
CL3
O1
C1
C7
CL2
C6
C5
CL1
C4
C3
O2
C2
C8
C9
C21
C20
C10
C11
C12
C13
C14
C15
C16
C17
C18
C19
N1

Table S3: Optimized structure (Cartesian coordinates, Å) of **7a** isomers calculated by the PBE0/6-311+ G(d,p) method in the gas phase.

**7a (NH)**				**7a (OH)**			
	X	Y	Z		X	Y	Z
1 C	5.133779	0.740503	-0.465361	1 C	5.127637	0.763631	-0.452105
2 C	4.718896	2.044618	-0.836623	2 C	4.711678	2.098690	-0.696011
3 C	3.405569	2.426372	-0.768508	3 C	3.401830	2.473968	-0.568939
4 C	2.484595	1.466857	-0.321527	4 C	2.474759	1.486270	-0.191937
5 C	2.821386	0.186792	0.047923	5 C	2.824289	0.175580	0.054802
6 C	4.175199	-0.220715	-0.002879	6 C	4.173744	-0.231813	-0.058190
7 C	0.492028	0.537547	0.247733	7 C	0.536502	0.553689	0.314612
8 N	1.110732	1.635309	-0.183904	8 N	1.105218	1.680403	-0.032428
9 C	-0.918267	0.509242	0.335495	9 C	-0.916949	0.517657	0.388851
10 C	-4.075495	0.970710	0.255628	10 C	-4.059442	1.001378	0.128521
11 C	-3.938018	-0.373637	-0.292193	11 C	-3.927985	-0.381749	-0.283608
12 C	-2.826487	-1.106133	-0.105115	12 C	-2.823054	-1.119662	-0.029471
13 C	-1.621048	-0.717034	0.719829	13 C	-1.621388	-0.708613	0.775444
14 O	-1.226861	-1.512804	1.546813	14 O	-1.207729	-1.482650	1.612700
15 C	-1.649466	1.737300	0.090656	15 C	-1.628780	1.706081	0.172635
16 C	-3.081180	1.853713	0.422232	16 C	-3.053509	1.880065	0.314594
17 O	-1.102808	2.801718	-0.252736	17 O	-1.024939	2.843641	-0.060584
18 Cl	-2.666102	-2.681005	-0.778888	18 Cl	-2.722685	-2.756263	-0.547448
19 Cl	-5.260031	-0.986869	-1.216495	19 Cl	-5.259195	-1.080153	-1.131000
20 Cl	-5.679732	1.461429	0.707032	20 Cl	-5.668564	1.602116	0.365924
21 H	0.520277	2.459090	-0.388976	21 H	-0.003660	2.631397	-0.116362
22 C	1.562251	-0.534793	0.507250	22 C	1.572748	-0.568216	0.479452
23 C	1.288335	-1.802797	-0.315092	23 C	1.254213	-1.763236	-0.429965
24 H	1.174077	-1.560648	-1.374884	24 H	1.127402	-1.440472	-1.466863
25 H	2.124108	-2.498028	-0.224136	25 H	2.071905	-2.485731	-0.407442
26 H	0.391642	-2.313645	0.032686	26 H	0.350261	-2.277994	-0.104318
27 C	1.677686	-0.818164	2.019783	27 C	1.728202	-0.972892	1.961057
28 H	2.539811	-1.462043	2.205758	28 H	2.593101	-1.630484	2.071636
29 H	1.834413	0.112262	2.572751	29 H	1.903759	-0.089176	2.580969
30 H	0.774513	-1.302289	2.385794	30 H	0.836022	-1.482619	2.317810
31 C	4.647267	-1.504856	0.371821	31 C	4.643940	-1.547628	0.187012
32 C	6.497694	0.373285	-0.541109	32 C	6.486722	0.397916	-0.588945
33 C	5.978619	-1.823071	0.287273	33 C	5.971136	-1.864131	0.046395
34 C	6.916650	-0.879242	-0.175790	34 C	6.904727	-0.885320	-0.347579
35 H	7.966242	-1.145707	-0.238533	35 H	7.951328	-1.149336	-0.456120
36 H	3.950897	-2.249349	0.736101	36 H	3.948272	-2.318288	0.494217
37 H	6.313352	-2.812431	0.581548	37 H	6.305715	-2.877941	0.240815
38 H	7.212813	1.109794	-0.895640	38 H	7.200001	1.159678	-0.889909
39 H	5.471474	2.747567	-1.179765	39 H	5.462466	2.826177	-0.988880
40 H	3.085582	3.423749	-1.048799	40 H	3.077005	3.492076	-0.752420
41 H	-3.337219	2.843792	0.784215	41 H	-3.327318	2.905075	0.540088

Table S4: Optimized structure (Cartesian coordinates, Å) of **7b** isomers calculated by the PBE0/6-311+ G(d,p) method in the gas phase.

**7b (NH)**				**7b (OH)**			
	X	Y	Z		X	Y	Z
1 C	5.069078	-1.474948	-0.117822	1 C	5.063888	-1.488305	-0.242906
2 C	5.172173	-0.072351	-0.301359	2 C	5.167685	-0.076451	-0.338218
3 C	4.006758	0.748304	-0.212961	3 C	4.003997	0.735917	-0.184662
4 C	2.799115	0.071939	0.057713	4 C	2.789751	0.052484	0.056370
5 C	2.712335	-1.285340	0.236668	5 C	2.713423	-1.318793	0.147305
6 C	3.863366	-2.079654	0.151603	6 C	3.860578	-2.109113	0.001546
7 C	0.586682	-0.297093	0.427993	7 C	0.638190	-0.310979	0.418974
8 N	1.525187	0.619493	0.183885	8 N	1.531752	0.627650	0.218511
9 C	-0.774216	0.077286	0.466980	9 C	-0.762111	0.076278	0.471670
10 C	-3.657207	1.431663	0.346529	10 C	-3.612213	1.487486	0.253905
11 C	-3.866099	0.178915	-0.370198	11 C	-3.871211	0.185370	-0.327083
12 C	-3.022905	-0.861707	-0.249053	12 C	-3.040001	-0.870120	-0.165249
13 C	-1.812614	-0.938449	0.651948	13 C	-1.804326	-0.931117	0.688290
14 O	-1.702970	-1.913991	1.365325	14 O	-1.661112	-1.893252	1.413021
15 C	-1.108451	1.484635	0.377329	15 C	-1.086908	1.438012	0.402098
16 C	-2.470188	1.967398	0.664635	16 C	-2.403181	2.005653	0.552976
17 O	-0.256704	2.378678	0.213775	17 O	-0.165064	2.364255	0.320016
18 Cl	-3.274455	-2.324919	-1.117887	18 Cl	-3.400264	-2.388924	-0.886130
19 Cl	-5.242930	0.086408	-1.406686	19 Cl	-5.313269	0.014471	-1.259661
20 Cl	-5.086688	2.307471	0.802867	20 Cl	-4.985852	2.501582	0.556336
21 H	1.202459	1.596970	0.086023	21 H	0.748813	1.864610	0.245897
22 H	3.808468	-3.154491	0.292568	22 H	3.807025	-3.191085	0.075020
23 H	5.972106	-2.072416	-0.193939	23 H	5.965449	-2.080431	-0.367362
24 C	1.281611	-1.660067	0.561672	24 C	1.288925	-1.701209	0.466757
25 C	0.743941	-2.713171	-0.414900	25 C	0.712687	-2.677250	-0.565551
26 H	0.755993	-2.341043	-1.442474	26 H	0.710048	-2.238215	-1.566777
27 H	1.385161	-3.597810	-0.370399	27 H	1.333866	-3.576866	-0.595784
28 H	-0.268487	-3.021263	-0.156309	28 H	-0.301120	-2.982655	-0.305631
29 C	1.240905	-2.164494	2.018857	29 C	1.273760	-2.303531	1.887093
30 H	1.881316	-3.047154	2.102077	30 H	1.903963	-3.197601	1.896569
31 H	1.625998	-1.404457	2.704233	31 H	1.685198	-1.595602	2.611827
32 H	0.223515	-2.424481	2.304738	32 H	0.261756	-2.570855	2.183450
33 H	-2.465958	2.940200	1.144685	33 H	-2.377392	3.032453	0.901523
34 C	6.412010	0.553939	-0.572951	34 C	6.406360	0.562892	-0.583582
35 C	4.116409	2.145419	-0.393660	35 C	4.114671	2.140384	-0.278322
36 C	6.494212	1.911919	-0.744338	36 C	6.488839	1.929162	-0.669984
37 C	5.337489	2.713930	-0.653507	37 C	5.333797	2.723896	-0.515784
38 H	3.231852	2.770971	-0.325200	38 H	3.225794	2.750474	-0.159709
39 H	5.415755	3.787203	-0.790502	39 H	5.412547	3.803780	-0.586218
40 H	7.302900	-0.062970	-0.642650	40 H	7.295335	-0.049533	-0.702101
41 H	7.452807	2.376070	-0.950903	41 H	7.445815	2.404781	-0.857687

Table S5: Optimized structure (Cartesian coordinates, Å) of **8a** isomers calculated by the PBE0/6-311+ G(d,p) method in the gas phase.

**8a (NH)**				**8a (OH)**			
	X	Y	Z		X	Y	Z
1 C	5.332975	0.752213	-0.546963	1 C	5.300700	0.817420	-0.556830
2 C	4.842979	1.901251	-1.217696	2 C	4.785989	2.035240	-1.073183
3 C	3.502872	2.175061	-1.286602	3 C	3.446309	2.313655	-1.048316
4 C	2.634001	1.264084	-0.667694	4 C	2.591684	1.344402	-0.495284
5 C	3.044057	0.132274	-0.005993	5 C	3.037183	0.144729	0.017160
6 C	4.425002	-0.161516	0.083420	6 C	4.418242	-0.159306	0.011947
7 C	0.687761	0.322596	0.033163	7 C	0.720495	0.366890	0.163822
8 N	1.244183	1.339602	-0.624127	8 N	1.205392	1.445899	-0.396315
9 C	-0.714679	0.218519	0.141803	9 C	-0.723207	0.251692	0.283875
10 C	-3.886301	0.508713	0.158329	10 C	-3.871996	0.481804	0.050361
11 C	-3.656594	-0.830720	-0.377008	11 C	-3.585153	-0.872629	-0.398285
12 C	-2.520203	-1.499530	-0.139081	12 C	-2.459836	-1.530376	-0.062036
13 C	-1.357666	-0.984103	0.681037	13 C	-1.349132	-0.979239	0.788354
14 O	-0.951507	-1.662028	1.599051	14 O	-0.933855	-1.634002	1.716932
15 C	-1.520206	1.337135	-0.306607	15 C	-1.502897	1.358175	-0.073204
16 C	-2.932541	1.456927	0.203011	16 C	-2.945610	1.460937	0.194692
17 O	-1.075673	2.300755	-0.933857	17 O	-0.976837	2.451646	-0.530180
18 Cl	-2.225933	-3.068753	-0.776229	18 Cl	-2.166566	-3.145032	-0.569878
19 Cl	-4.919306	-1.513759	-1.335890	19 Cl	-4.786930	-1.640298	-1.371466
20 Cl	-5.476222	0.818516	0.755019	20 Cl	-5.526245	0.811888	0.408368
21 H	0.625892	2.054577	-1.026464	21 H	0.070892	2.270412	-0.603916
22 C	1.818520	-0.583212	0.543903	22 C	1.835953	-0.612649	0.550108
23 C	1.690765	-2.004126	-0.028925	23 C	1.654005	-1.975173	-0.135213
24 H	1.604926	-1.975551	-1.118308	24 H	1.515351	-1.853808	-1.212956
25 H	2.581268	-2.585014	0.215782	25 H	2.541440	-2.592416	0.014545
26 H	0.827028	-2.523211	0.383786	26 H	0.800759	-2.515177	0.274703
27 C	1.855472	-0.559523	2.085042	27 C	1.953504	-0.729589	2.083802
28 H	2.736113	-1.097765	2.441194	28 H	2.849118	-1.299139	2.340788
29 H	1.925806	0.468878	2.450094	29 H	2.050837	0.261508	2.535653
30 H	0.959539	-1.024112	2.492911	30 H	1.078998	-1.225603	2.500649
31 C	4.969534	-1.288081	0.751420	31 C	4.985364	-1.353506	0.526854
32 C	6.722683	0.495064	-0.486903	32 C	6.689205	0.552130	-0.591553
33 C	6.323552	-1.502490	0.790140	33 C	6.338233	-1.573993	0.477504
34 C	7.212853	-0.606106	0.164802	34 C	7.201920	-0.615515	-0.088476
35 H	8.281259	-0.789341	0.203058	35 H	8.269810	-0.803341	-0.122076
36 H	4.310705	-1.992123	1.243689	36 H	4.344941	-2.105682	0.970558
37 H	6.714360	-2.371820	1.308766	37 H	6.747770	-2.495230	0.878935
38 Cl	-3.260392	3.014654	0.855062	38 Cl	-3.427753	3.035364	0.703884
39 H	7.399587	1.193651	-0.969817	39 H	7.348086	1.298085	-1.026279
40 H	5.558608	2.572475	-1.681901	40 H	5.484860	2.752059	-1.493184
41 H	3.125571	3.054558	-1.796399	41 H	3.046583	3.242620	-1.439260

Table S6: Optimized structure (Cartesian coordinates, Å) of **8b** isomers calculated by the PBE0/6-311+ G(d,p) method in the gas phase.

	**8b (NH)**				**8b (OH)**		
	X	Y	Z		X	Y	Z
1 C	5.332260	-1.353617	0.283868	1 C	5.313482	-1.400233	0.041366
2 C	5.349437	-0.028216	-0.220110	2 C	5.317864	-0.026927	-0.315025
3 C	4.127323	0.694752	-0.374001	3 C	4.094872	0.708807	-0.331875
4 C	2.956206	0.005097	0.001646	4 C	2.928597	-0.008888	0.017627
5 C	2.951440	-1.276854	0.488598	5 C	2.946905	-1.341248	0.361602
6 C	4.157115	-1.975421	0.636469	6 C	4.151824	-2.055520	0.379850
7 C	0.756095	-0.439513	0.372305	7 C	0.798349	-0.461410	0.416680
8 N	1.641547	0.464256	-0.053613	8 N	1.627445	0.488497	0.056534
9 C	-0.624992	-0.161252	0.342029	9 C	-0.620989	-0.159156	0.433622
10 C	-3.586855	0.975609	0.110609	10 C	-3.536769	1.006261	0.047565
11 C	-3.639617	-0.298981	-0.601237	11 C	-3.602152	-0.312620	-0.564234
12 C	-2.756630	-1.279062	-0.364862	12 C	-2.746001	-1.306804	-0.260232
13 C	-1.614307	-1.206553	0.623527	13 C	-1.607551	-1.211145	0.715573
14 O	-1.506051	-2.082332	1.453315	14 O	-1.470379	-2.072376	1.554458
15 C	-1.047792	1.185264	0.014396	15 C	-1.017396	1.162558	0.196775
16 C	-2.438402	1.618081	0.393979	16 C	-2.391155	1.650454	0.382263
17 O	-0.286636	2.065335	-0.395158	17 O	-0.157293	2.099131	-0.058486
18 Cl	-2.803450	-2.779134	-1.203046	18 Cl	-2.881998	-2.866289	-0.967890
19 Cl	-4.899673	-0.487421	-1.766295	19 Cl	-4.876016	-0.578905	-1.699026
20 Cl	-5.110193	1.629390	0.592434	20 Cl	-5.057218	1.764418	0.342824
21 H	1.280610	1.370025	-0.377066	21 H	0.797396	1.623896	-0.090823
22 H	4.168174	-2.990315	1.021074	22 H	4.174800	-3.106261	0.652210
23 H	6.277110	-1.877674	0.387722	23 H	6.258608	-1.934635	0.042926
24 C	1.531471	-1.687432	0.816936	24 C	1.542638	-1.768399	0.715623
25 C	1.133590	-2.949279	0.038444	25 C	1.076335	-2.948097	-0.146464
26 H	1.197374	-2.783257	-1.039971	26 H	1.079980	-2.688219	-1.208359
27 H	1.826728	-3.754824	0.295801	27 H	1.762543	-3.787754	-0.004688
28 H	0.127137	-3.279039	0.292924	28 H	0.078508	-3.283222	0.137032
29 C	1.415030	-1.898161	2.338826	29 C	1.505196	-2.115676	2.217713
30 H	2.094162	-2.702465	2.635534	30 H	2.188483	-2.949112	2.403883
31 H	1.707199	-0.994410	2.880421	31 H	1.838999	-1.266696	2.820490
32 H	0.395550	-2.167787	2.609621	32 H	0.500036	-2.399485	2.524411
33 C	6.556916	0.614675	-0.583587	33 C	6.512851	0.648470	-0.660680
34 C	4.151260	2.015030	-0.877085	34 C	4.104623	2.075336	-0.686887
35 C	6.555383	1.896335	-1.070555	35 C	6.497260	1.976593	-1.002253
36 C	5.343094	2.602127	-1.218209	36 C	5.283776	2.695484	-1.015216
37 H	3.224387	2.568161	-0.993131	37 H	3.171039	2.627407	-0.696754
38 H	5.355182	3.616034	-1.603676	38 H	5.285490	3.745728	-1.287577
39 H	7.490419	0.071821	-0.469842	39 H	7.447131	0.094934	-0.650608
40 H	7.489917	2.374261	-1.344683	40 H	7.421596	2.480581	-1.264544
41 Cl	-2.447356	3.114805	1.241813	41 Cl	-2.460693	3.231499	1.065182
